# Microbiome changes through the ontogeny of the marine sponge *Crambe crambe*

**DOI:** 10.1186/s40793-024-00556-7

**Published:** 2024-03-11

**Authors:** Marta Turon, Madeline Ford, Manuel Maldonado, Cèlia Sitjà, Ana Riesgo, Cristina Díez-Vives

**Affiliations:** 1 Department of Biodiversity and Evolutionary Biology, Museo Nacional de Ciencias Naturales (MNCN-CSIC), c/José Gutiérrez Abascal 2, 28006 Madrid, Spain; 2 Department of Life Sciences, The Natural History Museum, Cromwell Road, London, SW7 5BD UK; 3 Department of Marine Ecology, Centre d’Estudis Avançats de Blanes (CEAB-CSIC), c/Accés a la Cala St. Francesc, 14, 17300 Blanes, Spain; 4 Department of Systems Biology, Centro Nacional de Biotecnología, c/Darwin, 3, 28049 Madrid, Spain

**Keywords:** Demospongiae, Porifera, Symbionts, Life cycle, 16S rRNA gene, Vertical transmission

## Abstract

**Background:**

Poriferans (sponges) are highly adaptable organisms that can thrive in diverse marine and freshwater environments due, in part, to their close associations with internal microbial communities. This sponge microbiome can be acquired from the surrounding environment (horizontal acquisition) or obtained from the parents during the reproductive process through a variety of mechanisms (vertical transfer), typically resulting in the presence of symbiotic microbes throughout all stages of sponge development. How and to what extent the different components of the microbiome are transferred to the developmental stages remain poorly understood. Here, we investigated the microbiome composition of a common, low-microbial-abundance, Atlantic-Mediterranean sponge, *Crambe crambe*, throughout its ontogeny, including adult individuals, brooded larvae, lecithotrophic free-swimming larvae, newly settled juveniles still lacking osculum, and juveniles with a functional osculum for filter feeding.

**Results:**

Using 16S rRNA gene analysis, we detected distinct microbiome compositions in each ontogenetic stage, with variations in composition, relative abundance, and diversity of microbial species. However, a particular dominant symbiont, Candidatus *Beroebacter blanensis,* previously described as the main symbiont of *C. crambe*, consistently occurred throughout all stages, an omnipresence that suggests vertical transmission from parents to offspring. This symbiont fluctuated in relative abundance across developmental stages, with pronounced prevalence in lecithotrophic stages. A major shift in microbial composition occurred as new settlers completed osculum formation and acquired filter-feeding capacity. Candidatus *Beroebacter blanensis* decreased significatively at this point. Microbial diversity peaked in filter-feeding stages, contrasting with the lower diversity of lecithotrophic stages. Furthermore, individual specific transmission patterns were detected, with greater microbial similarity between larvae and their respective parents compared to non-parental conspecifics.

**Conclusions:**

These findings suggest a putative vertical transmission of the dominant symbiont, which could provide some metabolic advantage to non-filtering developmental stages of *C*. *crambe*. The increase in microbiome diversity with the onset of filter-feeding stages likely reflects enhanced interaction with environmental microbes, facilitating horizontal transmission. Conversely, lower microbiome diversity in lecithotrophic stages, prior to filter feeding, suggests incomplete symbiont transfer or potential symbiont digestion. This research provides novel information on the dynamics of the microbiome through sponge ontogeny, on the strategies for symbiont acquisition at each ontogenetic stage, and on the potential importance of symbionts during larval development.

**Supplementary Information:**

The online version contains supplementary material available at 10.1186/s40793-024-00556-7.

## Background

Sponges (phylum Porifera) are considered among the earliest diverging metazoans, dating back at least to the Precambrian [1, 2]. Porifera can be found in almost all aquatic habitats, from freshwaters to the deep sea, coral reefs and even the intertidal [3, 4]. Porifera, despite being perceived as simplistic in their morphology and function [5], play essential roles in marine ecosystems [6, 7], having a significant effect on ecosystem provisions [8] and nutrient recycling [9, 10], and increasing biodiversity within benthic communities [4, 11, 12]. Their ability to function in multiple and varied environments suggests that sponges are plastic organisms [7, 13], referring to the concept of adaptive plasticity whereby organisms are able to cope with a number of different environmental conditions by being adaptable [14]. Symbiosis between Porifera and a wide range of prokaryotic partners is emerging as one of the reasons why sponges can exist in many different types of environments [15], with their varying microbiome composition promoting this adaptive plasticity and allowing them to survive in a plethora of environmental conditions [16, 17]. Microbiomes are involved in important biological processes that benefit both the microbes themselves and the sponge host [18, 19], including production of chemicals to deter predators and prevent disease and the processing of inorganic nutrients, including carbon and nitrogen.

In addition to nonspecific components of the microbiome, sponges also present species-specific microbial communities, which are singular to a sponge species [20–23], referred to as ‘host specificity’ [24]. This “conserved” component of the microbiome needs to be passed on with fidelity. The most extended pathway to ensure fidelity in transmission is via reproductive stages, that is, from adult sponges to gametes, embryos or larvae (i.e., vertical transmission) [25–28]. Some symbionts included in the different reproductive stages may present a nutritional role, and eventually be digested, therefore, a conservative definition of vertically transmitted symbionts would refer to microorganisms that persist throughout the entire gametogenic and/or embryonic development of their host [26, 29]. However, there is also evidence of horizontal acquisition for some conserved members of the microbiome [25, 30, 31] recruited from the environment [32, 33]. For instance, *Petrosia ficiformis* appears to rely exclusively on horizontal acquisition since gametes and early embryos of this sponge are found to be free of symbionts [34]. Our current understanding is that the sponge microbiome is acquired by a mix of both strategies [26, 29, 35]. However, the interplay between vertical and horizontal transmission during sponge ontogeny is poorly understood in most cases.

During the onset of reproductive processes, microbes can be vertically transmitted during oogenesis, either through phagocytosis by oocytes [36] or transmitted via nurse cells [37]. In oviparous sponges, embryonic development occurs in seawater columns after external fertilization [34], facilitating the acquisition of microbes from the surrounding environment. In the case of viviparous sponges, microbial incorporation from the parental sponge continues during embryogenesis and larval development, facilitated by processes such as phagocytosis [30, 37], cleavage furrows [38], or radiating cytoplasmic bridges [39]. In both cases, the free-swimming larval stage is always lecithotrophic, relying exclusively on the energy obtained from the yolk originally provisioned within the egg [40]. Once the larval energy reserves (i.e., yolk) are depleted and the correct signals for settlement are interpreted, larvae will settle, and after a few days, they will form an osculum to start pumping and filtering for feeding [41]. The formation of this osculum is the step completing the aquiferous system deployment and ensures a water current for both acquisition of particulate food and removal of waste products away from the sponge.

Studies on the microbiome composition of adults and larvae have demonstrated that the microbiome of sponge larvae typically constitutes a random subset of the microbial community present in the mother sponge [27, 30, 42–44]. However, limited research has explored changes occurring during later developmental stages, such as settlers and early juveniles, which can impact the fidelity and persistence of vertically transmitted symbionts [30]. For instance, in the case of the high microbial abundance (HMA) sponge *Carteriospongia foliascens* [45], deep-sea viviparous sponges [46], and the oviparous sponge *Iantella basta* [44], the authors found highly similar microbiome communities among adults, embryos, larvae, and early recruits, indicating substantial vertical acquisition with high fidelity of the microbiome. In contrast, low microbial abundance (LMA) sponge species, such as *Tedania* sp., exhibited significant differences in microbiome composition across seven ontogenetic stages, with only a few common microbial taxa present in small proportions across all stages [47].

*Crambe crambe*, a demosponge belonging to the order Poecilosclerida and family *Crambeidae,* is a LMA sponge with a microbial community dominated by a single bacterial species [48]. This symbiont was initially classified as *Betaproteobacteria* [48], but later identified as Ca. *Beroebacter blanensis*, a constituent of a novel bacterial order, Candidatus (Ca.) *Tethybacterales* [49]. This newly defined order falls within the *Gammaproteobacteria* class and it mainly consists of sponge symbionts [49]. *Crambe crambe* is a sublittoral, orange‒red, encrusting sponge, common in the Western Mediterranean Sea [50–53] and at the Canary [54] and Madeira (P. Wirtz, personal communication 2002) archipelagos in the Eastern Atlantic Ocean. A few reports signal its presence in the Eastern Mediterranean, where it seems to be less abundant [55, 56]. Ecologically, *C. crambe* is one of the best-studied sponges [57–59]. It has been referred to as a biological indicator species [60], whereby its presence indicates the quality of environmental conditions, reinforcing the idea that sponges play important ecological roles. *C. crambe* is a simultaneous hermaphrodite with internal fertilization [52, 61]. The sponge releases lecithotrophic parenchymella larvae for dispersal. Larvae swim in a slow corkscrew motion from hours to several days before settlement, which occurs spontaneously in laboratory conditions, allowing experimentation [52, 62–64].

In the present study, we investigated the microbiome composition of the marine sponge *C. crambe,* focusing on the patterns of microbiome diversity and variation over its ontogeny, including brooded pre-competent larvae, free-living larvae, and settled juveniles before and after developing a functional osculum. In all cases, brooded larvae, larvae, juveniles and adults were analysed individually (i.e., not by pooling). We employed 16S rRNA gene analysis, generating inferred amplicon sequence variants (ASVs), to elucidate the proportion of the adult sponge microbiome that is maintained through the different ontogenetic stages, as well as the differences in alpha and beta microbial diversity between stages. Only by considering a broad range of developmental stages and by analysing them independently we can gain a comprehensive understanding of the complexities underlying the processes of transmission and maintenance of symbiotic microbial communities in sponges.

### Methods

#### Sample collection and DNA extraction

**Fig. 1:**
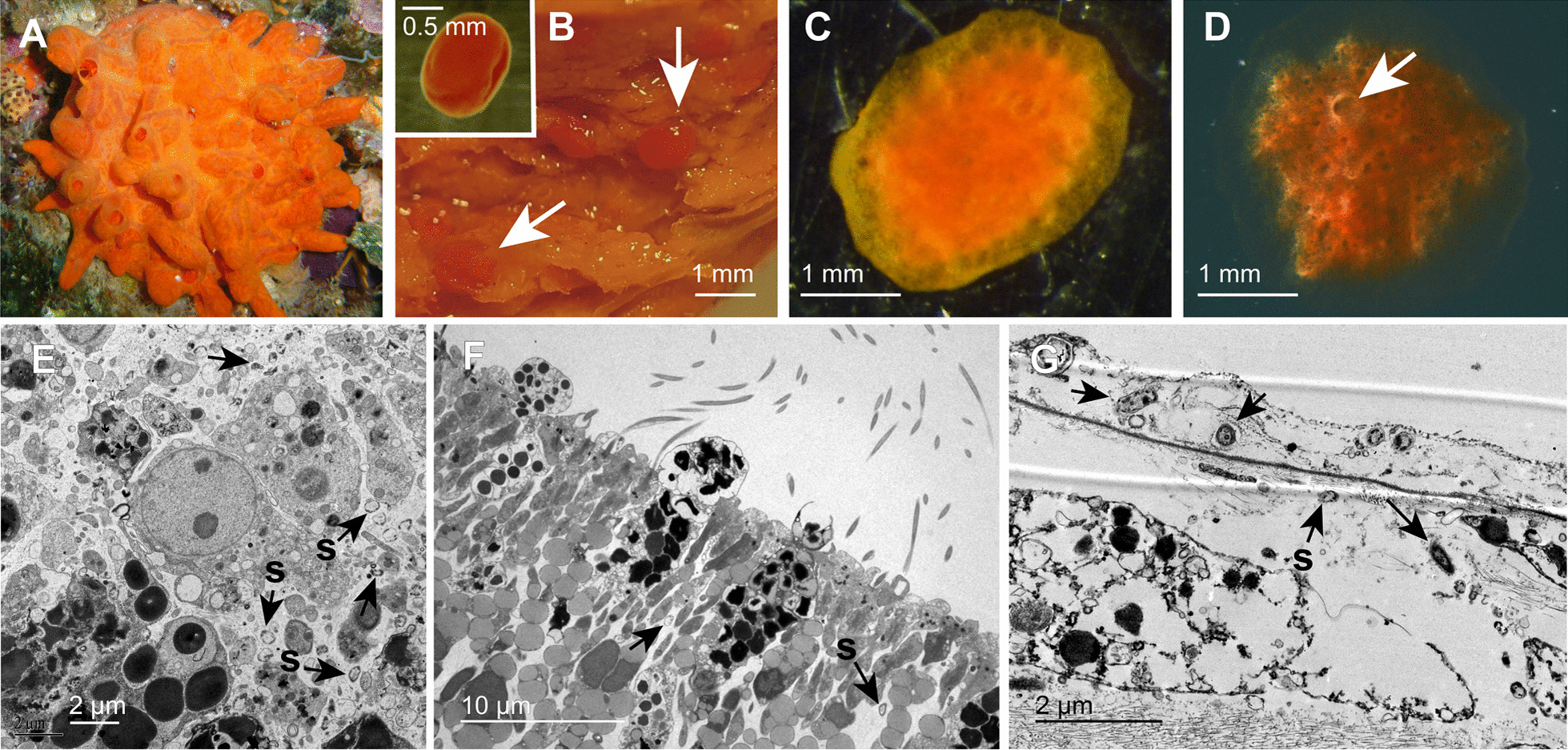
Pictures of all the *Crambe crambe* ontogenetic stages analysed (**A**–**D**) and ultrastructural images of their tissue and associated microbes E–G. **A** Adult individual of *Crambe crambe* in the field. **B** Brooded larvae (arrows) within the maternal tissue and free-swimming larva (inset). Scale bar: 1 mm for embryos and 0.5 mm for the free-swimming larva (inset). **C.** Settled juvenile without an osculum (JNO). Scale bar: 1 mm. **D** Settled juvenile with an osculum (JO). Note the osculum in the upper part of the juvenile sponge (arrow). Scale bar: 1 mm. **E** Mesohyl in the adult sponge showing a variety of symbionts (arrows), with a bacterium morphotype more abundant than the others (s). **F** Larval flagellated epithelium showing very few symbionts (arrows) in the intercellular medium, except for a common bacterial morphotype (s), which also occurrs in the adult (**E**). **G** Mesohyl of a juvenile with osculum showing few symbionts (arrows), with a common bacterium morphotype (s) similar to that occurring in adults and larvae (**E** and **F**)

Three reproductive individuals (containing brooded larvae) of *Crambe crambe* were sampled (ca. 3 cm^2^) on 9th August 2017 in Santa Anna, Blanes, Spain (41.6732, 2.8027). For DNA extraction, three pieces of tissue of ca. 5 mm^2^, from each individual were dissected, checking for the absence of brooded larvae in these tissue pieces. These constituted the three adult samples (AD), with three pseudo replicates per individual. At the same time, from each of the reproductive individuals collected, three to five brooded pre-competent larvae (BL) were excised, removing any adult tissue, and washed in sterile water. In addition, a total of thirty free-swimming larvae (FL) were collected from the surrounding water on the same day (9th August 2017) and the following day (10th August 2017). Unfortunately, the spawning adults of these FL were unknown. Yet, free-living larvae of *C. crambe* are easily recognisable by timing of release, colour, shape, and swimming pattern. Ten of those FL samples were directly preserved in RNAlater (Ambion) for further processing (five from Aug. 9th and five from Aug. 10th), and twenty others (exclusively from August 10th) were kept in a single aerated container at the aquarium facilities (LEOV, CEAB-CSIC) until settlement, which took approximately 48–72 h. The water in the container was collected from the sponge habitat and was not changed during the twenty days that the experiment lasted. Four juveniles with no-osculum (JNO) were sampled seven days after larvae were added to the container when they were settled but their osculum were still underdeveloped (17th August 2017). The rest of the settled juveniles were left in the container until they developed an osculum two days later. Then, four settled juveniles with an open osculum (JO) were collected immediately upon the completion of a functional pumping system (19th August) and four other juveniles were collected after ten days of pumping activity (30th August 2017). Eight other larvae were left in the container, but we did not sample them since the development of the osculum was not evident. All samples were preserved in RNAlater at 4 °C overnight and then frozen at -20 °C until further processing. Pictures of all developmental stages analysed are shown in Fig. 1. DNA was isolated per adult tissue, individual larva and individual juvenile using a NucleoSpin Tissue XS Micro kit for DNA from cells and tissue (Macherey–Nagel, Germany).

### Transmission electron microscopy

A piece of adult tissues, three free-swimming larvae, and three juvenile samples were also fixed and preserved in 2.5% glutaraldehyde in 0.2 M PBS and 1.4 M sodium chloride for ultramicroscopy purposes. After 24 h, samples were desilicified in hydrofluoric acid for 1.5 h, rinsed three times (10 min each time) in 0.4 M PBS and then in distilled water, post-fixed in 2% osmium tetroxide in PBS for 2 h, rinsed in distilled water and dehydrated in an ascending acetone series until finally embedded in Spurr’s resin. Ultrathin sections were obtained with an Ultracut Reichert-Jung ultramicrotome and mounted on gold grids and stained with 2% uranyl acetate for 30 min, followed by lead citrate for 10 min. Observations were conducted with a JEOL 1010 transmission electron microscope operating at 80 kV and fitted with a Gatan module for acquisition of digital images.

### 16S rRNA gene amplification

The 16S rRNA gene V4 region was amplified using the universal microbial primers 515F-Y [65] and 806R [66], which amplify both bacterial and archaeal members. DNA amplification was always performed in duplicate, using the PCRBIO HiFi Polymerase (PCR Biosystems Ltd) under the following conditions: 98 °C for 30 s, followed by 30 cycles of 98 °C for 9 s, 55 °C for 1 min, 72 °C for 1.5 min, and a final elongation at 72 °C for 10 min. Verification of PCR-products was accomplished by electrophoresis on an agarose gel. PCR products were purified with AgencourtAMPure XP Beads (Beckman Coulter Inc.), and libraries were prepared with the Nextera XT DNA Library Preparation Kit (Illumina Inc.). Next-generation, paired-end sequencing was performed on the Illumina MiSeq platform at the Natural History Museum of London sequencing facility using v3 chemistry (2 × 300 bp).

### Microbiome pipeline analysis

Raw paired reads were imported into Mothur (v.1.48.0) [67], and an adaptation of the MiSeq SOP protocol was followed [68]. Briefly, primer sequences were removed, and sequence contigs were built from overlapping paired reads. Sequences with > 0 N bases or with > 15 homopolymers were discarded. Unique sequences were aligned against the Silva reference data set (release 132) [69], and poorly aligned sequences (deviants from the expected start and end positions) were removed. Unoise3 [70], which is implemented within Mothur, was used for denoising (i.e., error correction) of unique aligned sequences to infer amplicon sequence variants (ASVs), allowing one mismatch per 100 bp. Any singletons remaining at this stage were removed. Reference-based chimera checking was conducted using UCHIME with the Silva reference data set and parameter min = 0.3. ASVs were classified using the Silva database v.132 [69], with a cut-off value of 80. ASVs classified as eukaryotic, chloroplast, mitochondria or unknown were discarded. A specific sequence similarity search using the Basic Local Alignment Search Tool (Blast), available on the National Center for Biotechnology Information (NCBI) platform, was performed for the most dominant ASV (ASV00001) to assess whether or not it belonged to the already described dominant symbiont for *C. crambe.*

### Statistical design and analysis

All the statistical analyses were performed in R v.3.6.1 [71]. Beta diversity analysis was calculated using the Bray–Curtis dissimilarity coefficient based on the log2 transformed relative abundance of the original ASV table. Principal coordinates analysis (PCoA), requiring the “vegan” v2.5–6 package [72] and corresponding “cmdscale” function, allowed visualization of distance matrices between microbiome ASVs present in the different *C. crambe* individuals/developmental stages. To run the statistics, *individual* factor (for adults and brooded larvae) was nested within sample stage. Homogeneity of dispersion between groups was checked using the *permutest* function of the “vegan” package prior to conducting non-parametric Permutational Analysis of Variance (PERMANOVA) using the *adonis* function to examine variation in microbial composition between ontogenetic stages. Using the same method, the time effect was also tested for free-swimming larvae (FL) and juveniles with osculum (JO) samples.

For alpha diversity (Shannon and Inverse Simpson indexes), we used a rarefied ASV dataset to the minimum reads’ threshold of 17,775. Normality was checked using *Shapiro* test prior to perform an analysis of variance (ANOVA) to compare alpha diversity among stages, and Tukey’s honestly significant difference (HSD) was used for addressing pairwise comparisons. Again, the effect of time was tested for FL and JO samples.

To test parents to offspring similarity we calculated the Bray–Curtis dissimilarity matrix and Jaccard distance matrix of the adults and brooded larvae subset. We then performed ANOVA tests on the Bray–Curtis and Jaccard distances to find significant differences between sibling larvae (originating from the same individual) and non-sibling larvae (from different parents), and between brooded larvae and their related and unrelated adults. Core members of each individual pair (adult-brooded larvae) were assessed as ASVs present in all the replicates from the same individual and the number of shared and exclusive ASV members was represented using Venn diagram and Upset plot.

The core community of each ontogenetic stage was calculated considering ASVs present in 70% and 100% of the samples within each stage. Upset plots from the *ComplexUpset* package [73] and Venn diagrams from the *eulerr* package [74] were employed to visualize the number of shared ASVs between the different ontogenetic stages analysed using the cores at 70%. For clarity, samples belonging to the same stages were pooled together, regardless of the time. Separate analysis considering time for FL and JO samples are presented in the Supplementary Material. Variation of the relative abundance of the ASV00001 across stages was evaluated using generalized linear mixed models (GLMM), with the *individual* factor nested within stage. To test for significant differences, analysis of variance (ANOVA) was performed, followed by the post-hoc Tukey’s test for all models, using the packages *car* [75] and *emmeans* [76]*.*

Differential Abundance analysis using generalized linear model was performed in the R package “EdgeR” v.3.26.8 [77] to discern variations in the abundance of particular microbiome ASVs across different ontogenetic groups. We used subsets of the specific comparisons and ASVs were filtered by a minimum relative abundance of 0.001% in at least one sample. We considered individuals as blocking factors in the model design when pseudo replication between samples occurred (AD and BL). For representation purposes, only comparisons between consecutive stages are shown in the main text. However, comparisons considering different times for FL and JO are shown in the Supplementary material.

## Results

### Microbial composition across *Crambe crambe* ontogeny

**Fig. 2:**
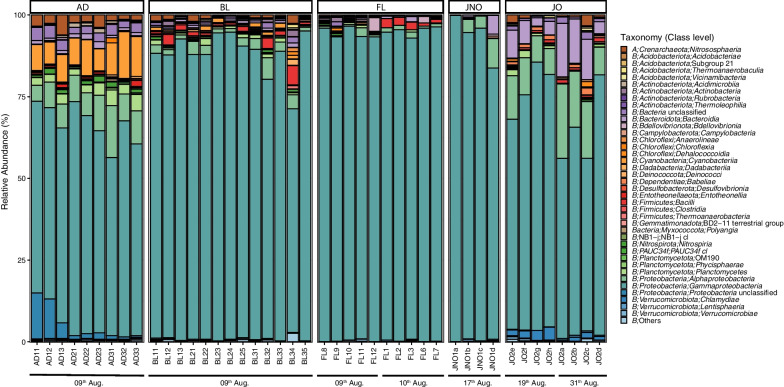
Microbial Taxonomic composition in *C. crambe* during different developmental stages: Barplots showing the taxonomic composition at class level for each sample, grouped by ontogenetic stage. In the legend; **A** Archaea, **B** Bacteria

Very few symbionts were observed in the adult and juvenile mesohyl, as well as in the larvae (Fig. 1). While transmission electron microscopy revealed a bacterium morphotype being common in all stages (Fig. 1E–G), metabarcoding revealed a more diverse prokaryotic community. Within the 44 *C. crambe* samples sequenced, a total of 32,976 ASVs were identified (4,941 ASVs when filtering ASVs with relative abundance (RA) < 0.01), with reads ranging from its highest value of 121,951, mean value of 69,741, and a minimum value of 32,097. A total of 43 different prokaryotic phyla were identified in our data set (Additional file 12), with *Proteobacteria* being the most abundant phylum across samples, with an average relative abundance (avgRA) of 88.05%, followed by *Bacteroidota* (2.47%) and *Cyanobacteria* (2.1%). At the class level, 113 different classes were identified (Additional file 13), with *Gammaproteobacteria* being the most dominant class, with an avgRA of 81.01%, followed by *Alphaproteobacteria* (6.02%) and *Bacteroidia* (2.46%) (Fig. 2).

Overall, the bacterial communities within the different life stages were characterized by the dominance of *Gammaproteobacteria*, which ranged from an avgRA of 61.78% in adults to a maximum of 94.43% in free-living larvae (Fig. 2, Additional file 13). Despite the high abundance of *Gammaproteobacteria*, clear differences in certain bacterial groups were identified across life stages (Fig. 2). For instance, adult samples showed high percentages of *Cyanobacteria* (9.48%) and *Planctomycetes* (3.18%), which had abundances below 1% in the other life stages. The class *Alphaproteobacteria* represented important proportions in adults and juveniles with osculum (8.9% and 13.27%, respectively) having lower avgRA values in the other life stages. All the non-feeding (i.e., lecithotrophic) stages were characterized by a high predominance of *Gammaproteobacteria* (avgRA > 85%), followed by *Alphaproteobacteria* (avgRA 2–4%), *Bacilli* (avgRA ~ 1% in the case of BL and FL) and *Bacteroidia* (avgRA ~ 1% in the case of JNO). Juveniles with open osculum had a distinctive composition, with many different classes, including high levels of *Alphaproteobacteria*, *Bacteroidia* and *Chlamydiae*. This pattern supports the idea of a varying microbiome composition across the different stages of ontogeny.

The most dominant ASV (ASV00001), classified here as *Gammaproteobacteria,* had 100% similarity with the sequence of the dominant symbiont reported for *C. crambe*, previously classified as *Betaproteobacteria* [48]. In a more recent metagenomic study, the metagenome-assembled-genome (MAG) of this symbiont was obtained and classified within a new *Gammaproteobacteria* order, the *Ca. Tethybacterales* [49]. This main *C. crambe* symbiont was named *Ca. Beroebacter blanensis* and corresponds to our ASV00001.

### Microbial diversity across *C. crambe* ontogeny

**Fig. 3:**
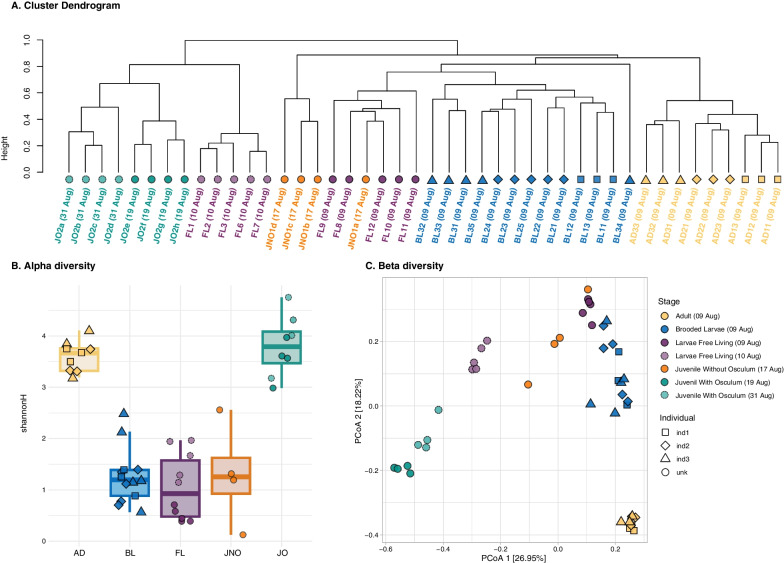
Alpha and Beta microbial diversity within different developmental stages of *C. crambe*: **A**. Cluster dendogram based on Bray–Curtis dissimilarity matrices of microbial communities in the ontogenetic cycle of the sponge *C. crambe*. Each colour depicts a developmental stage and sampling dates are indicated in brackets. **B**. Box plot showing alpha diversity measures of *C. crambe* ontogenetic stages using Shannon diversity index. **C.** Principal coordinate analysis (PCoA) plot based on Bray–Curtis dissimilarity index of the microbial composition across the different *C. crambe* ontogenetic phases (indicated by different colours). Shapes on the figure legend correspond to different adult individuals. Variation explained by the first two axes is indicated as %

Alpha diversity values for *C. crambe* were highly different across the ontogenetic stages, with significant differences revealed by Shannon and inverse Simpson diversity indexes (ANOVA, *p* < 0.001). The Shannon diversity index ranged from 0.12 in a juvenile with no osculum (JNO) to 4.75 in a juvenile with an open osculum (JO) (Additional file 14), while the inverse Simpson index ranged from 1.03 in a JNO sample to 24.1 in a JO sample (Additional file 14). Both indexes suggested that the microbial community of juveniles with filter-feeding capacity was the most diverse across the ontogeny, followed by adult samples (Fig. 3B). In contrast, stages without filtering capacity (BL, FL, JNO) had the lowest diversity values and, on average, showed similar diversity values between them (Fig. 3B). The Tukey pairwise test confirmed that adults and juveniles with osculum had significantly higher microbiome diversity than the remaining ontogenetic groups (Additional file 11). Moreover, we detected significant differences between sampling dates in alpha diversity metrics for FL larvae (ANOVA, *p* < 0.001), but not for JO (ANOVA, *p* > 0.05).

Beta diversity was calculated using Bray‒Curtis dissimilarities of log2 transformed relative abundances. Homogeneity of dispersion was checked using the *permutest* function of the vegan package for the ontogeny stage (*p* = 0.2), indicating similar dispersions among groups. The ordination of microbial community composition by group is shown in Fig. 3C, with the different ontogenetic stages separated along the first axis and the second axis separating adult samples from the juveniles and the other samples. Adult pseudo-replicates were pooled together for the statistical analysis once confirmed that no differences were detected among tissue replicates from the same individual (*adonis,* *p* > 0.05, Additional file 11). The results of the PCoA showed that the first two principal components combined explained 45% of the microbiome variation across *C. Crambe* developmental stages. The PERMANOVA test confirmed significant compositional differences between developmental stages for *C. crambe* (*adonis*, *p* < 0.001, Supplementary Material B). Moreover, significant differences between sampling dates were detected for FL and JO samples (Additional file 11).

The relationships between samples were summarized in a dendrogram (Fig. 3A), generated via hierarchical clustering based on the Bray‒Curtis dissimilarity matrix of the microbial ASVs. This clustering revealed two primary branches, with dissimilarity exceeding 80%. One cluster encompassed a group of juveniles with functional osculum (JO) and free-swimming larvae (FL 10th Aug.), while the other comprised the remaining *C. crambe* samples. This latter cluster included a group with FL (9th Aug.) and juveniles with no osculum (JNO), while brooded larvae (BL) and adult (AD) specimens made a cluster of their own. Moreover, subclusters corresponding to parental individuals (1–3) were observed for adults (pseudo-replicates from the same individual) and their corresponding offspring.

### Offspring similarity to parents and other conspecific adults

**Fig. 4:**
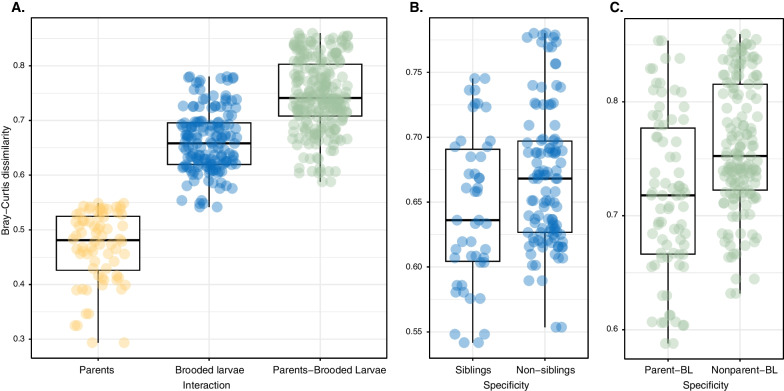
*Crambe crambe* Offspring similarity: Boxplots of Bray–Curtis distances of specified interactions: **A** Adult-Adult, Brooded larvae-Brooded larvae, Adult-Brooded larvae, **B** Brooded larvae comparing siblings between them and with non-siblings and **C** Parents and brooded larvae comparing adults and their own larvae, and adults with non-related larvae

We investigated microbiome similarity within individual adults, among brooded larvae originating from different individuals, and between adults with their own and foreign offspring using both Bray‒Curtis dissimilarity and the Jaccard index, producing very similar results. Overall, adults exhibited a higher degree of similarity to one another compared to larvae (Fig. 4A). For brooded larvae, those originating from the same individual (referred to as siblings) displayed significantly greater similarity (ANOVA, *p* < 0.05) when compared to larvae from other parents (Fig. 4B, Additional file 11). Additionally, when compared to their respective parents and other adults, the former case showed significantly higher similarity (Fig. 4C). Additional file 1 provides a more comprehensive analysis of the shared ASVs between parental individuals and their larval offspring. In terms of microbial richness, brooded larvae from adult 1 exhibited the highest value, also displaying the largest number of shared ASVs with their parental sponge (73 ASVs). This was followed by brooded larvae from adult 2, which shared 28 ASVs with their parental sponge, and brooded larvae from adult 3, which shared 22 ASVs. When examining the shared ASVs between different adult-larvae individual pairs, we observed that pairs corresponding to the same parent had the highest number of shared taxa, supporting the above mentioned idea of a higher degree of similarity in the microbiomes between adults and their respective larvae than to larvae produced by others. Furthermore, each larva had a unique set of ASVs that did not originate from their respective parental adults. These unique ASVs ranged from 5 to 21 among individuals, indicating the potential acquisition of microbes from sources other than their parents.

### Core microbiome across *Crambe crambe* ontogeny

Each ontogenetic stage exhibited a distinctive microbial composition, characterized by a core community that accounted for more than 60% of the relative abundance within each analysed stage (Additional file 15). Notably, both adults and juveniles with osculum displayed a larger number of core community members, with over 200 ASVs comprising their respective cores (core at 70%). In contrast, the core community of free-living larvae consisted of only 4 ASVs (considering 70% of the samples). Despite the small number, these ASVs exhibited high abundance, collectively representing 77% of the average relative abundance within this particular stage. There were 40 ASVs consistently found in all brooded larvae (BL) replicates (Additional file 15), which accounted for 88% of the average relative abundance within the BL stage. This strong consistency across the 13 BL analysed, which developed from 3 different adult individuals, suggests that these ASVs could potentially represent faithful vertically transmitted symbionts at the sponge species level.

**Fig. 5:**
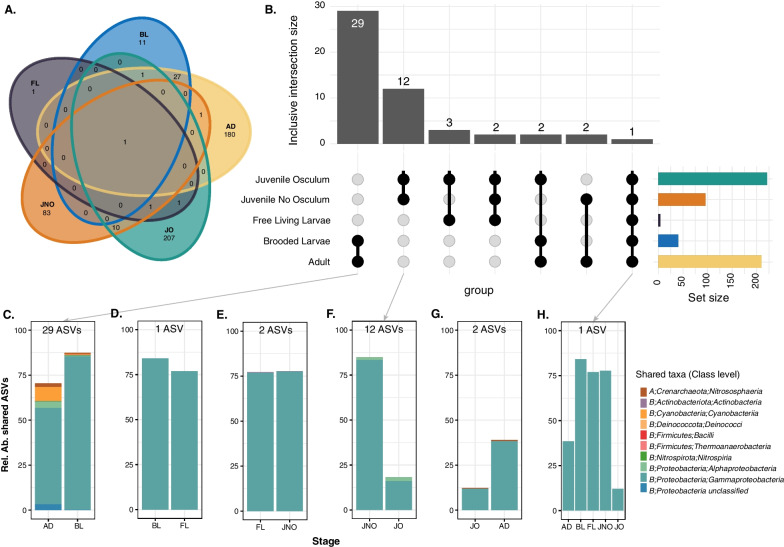
ASVs shared between ontogenetic stages in *C. crambe*. **A** Venn diagram showing all the interactions. **B** Upset plot showing inclusive intersections between consecutive stages analysed. Set size corresponds to core community values (assessed at 70% of replicates) and bars represent the size (no. ASVs) of the indicated intersections in the matrix ordered by decreasing values. **C**–**H** Relative abundance and composition of each consecutive developmental stages in *C. crambe*, and across all the stages (**H**). Taxonomic composition is indicated at class level. On top of each panel, number of shared ASVs that account for the plotted relative abundance in each intersection

These core community members (ASVs found in 70% of the replicates in each stage) were used to assess the number of shared and exclusive ASVs in each developmental stage (Fig. 5A, B). Each stage possessed a distinctive set of exclusive ASVs, present in 70% of the stage replicates, which were not found in the core microbiome of the other stages (Fig. 5A, Additional file 2). In this sense, the juveniles with functional osculum (221 core ASVs) exhibited the highest number of unique ASVs (207) and only 14 shared with other stages. This was closely followed by adult individuals (210 core ASVs, with 180 unique and 30 shared). The exclusive members represented over 40% of the average relative abundance in juveniles with functional osculum, while the relative abundances of the exclusive members of other stages were low (< 15%) (Additional file 2). A single ASV (ASV00001, Ca. *Beroebacter blanensis*) was found to be shared across all samples from the *C. crambe* reproductive cycle and dominated the microbial community of the sponge throughout all developmental stages, showing significant differences in its relative abundance across stages (Fig. 5H, Anova, *p-val* < 0.001, Supplementary Material D), and becoming especially abundant in the non-feeding stages (Fig. 5C–H). Similar results were obtained when considering the different sampling times for FL and JO, as shown in Additional file 3.

Very few microbes were observed within the tissues of *C. crambe* but, given that in our histological analysis a single bacterium morphotype was consistently found in the adult mesohyl, the larval epithelium and the juvenile mesohyl (Fig. 1E–G), this bacterium could represent the most abundant taxon shared across stages, Ca. *Beroebacter blanensis*. A similar analysis was performed to obtain the core microbiome shared at the order level (Additional file 4). The results showed a reduction in the number of exclusive taxa at each developmental stage, with only adults and all juveniles presenting exclusive taxa, not found in any of the other ontogenetic stages. Overall, 10 different bacterial orders were shared among ontogenetic stages and constituted over 60% of the average relative abundance in all cases (Additional file 4).

The composition and abundance of shared taxa between consecutive ontogenetic stages is visualized in Fig. 5C–H and the relative abundances of these shared ASVs are presented in Additional file 16. The shared ASVs ranged from 1 to 12% of the total ASVs in the comparisons. Although these shared ASVs represented relatively small proportions in terms of the overall ASV count, they represented important proportions in relative abundance. The stages with the highest number of shared ASVs were adults and brooded larvae, which shared 29 ASVs belonging to 9 different microbial classes (Fig. 5C, Additional file 16). Among the non-feeding stages, only ASV00001 was shared between brooded larvae and free-living larvae (FL) (Fig. 5D, Additional file 16), while 2 ASVs were shared between FL and juveniles without osculum (JNO) at a remarkably similar relative abundance across these stages (Fig. 5E, Additional file 16). Juveniles (without and with osculum) shared up to 12 ASVs from 3 different classes (Fig. 5F, Additional file 16). Following these comparisons, juveniles (JO) and AD shared only 2 ASVs (*Nitrosphaeria* and *Gammaproteobacteria*), albeit at varying abundances (Fig. 5G, Additional file 16). This suggests that although juvenile sponges began to develop the microbiome of adults, they still exhibited distinct microbial communities at the time of sampling.

### Differentially abundant ASVs

**Fig. 6:**
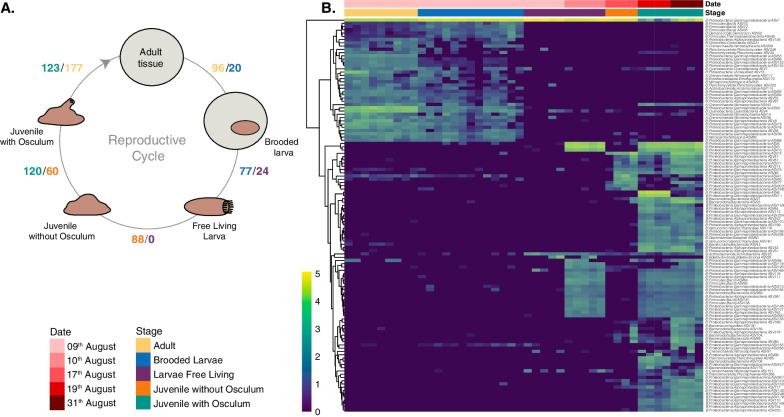
Differentially abundant microbes within *C. crambe* developmental stages **A** Differentially abundant (DA) ASVs across the reproductive cycle of the sponge *C. crambe*. Values represent the number of ASVs identified at higher relative abundances in the comparison between the consecutive ontogeny phases. Abundances and taxonomy of DA ASVs for each comparison can be found in Additional files 5–9 and 18. **B** Heatmap of the most abundant DA ASVs among the different ontogenetic stages of *C. crambe*, with log transformed abundances represented in the colour temperature bar. Microbial ASVs are organized according to a hierarchical clustering based on Bray–Curtis Dissimilarity matrices. Sponge samples (x-axis) are coloured according to stage and sampling date

Differential abundance (DA) analysis was conducted to identify specific ASVs that exhibited variations across different phases of the ontogenetic cycle in *C. crambe* (Fig. 6A, Additional file 17). A heatmap representation of the most abundant DA ASVs is shown in Fig. 6B. The comparison between adults (AD) and brooded larvae (BL) had 116 differentially abundant ASVs, with 96 ASVs found at higher abundances in the adults and 20 ASVs that increased their abundances in the BL (Additional file 17). These differences were primarily attributed to the higher abundance patterns of the ASV00009 (unclassified *Gammaproteobacteria*), the ASV0004 (*Synechoccales*) and the ASV0008 (unclassified *Alphaproteobacteria*) in adults individuals (Additional file 5, Additional file 18). ASV00001 was significantly different if all tissue samples from AD and BL were considered independently (not nested by individual), as their relative abundance changed from ca. 35–45% in adults to 70–80% in BL (Fig. 5H). In the following comparison (BL vs FL), brooded larvae presented larger numbers of ASVs that were more abundant in this stage than in free-living larvae (Fig. 6A, Additional file 6, Additional file 17). It is important to note that FL samples were collected on two different days, with larvae collected on the 10th of August presenting 42 ASVs at higher abundances than larvae collected on the 9th of August (Additional file 17, Additional file 18), characterized, among others, by an increase in ASV00003 (*Gammaprot*.; *Enterobacterales*; *Vibrio*), ASV00005 and ASV00013 (both *Gammaprot*.; *Pseudomonadales*; *Halomonas*), possibly captured somehow from the water (Additional file 6, Additional file 18).

The comparison of BL with FL from 9th Aug was characterized by a decrease in 16 ASVs from the BL stage, while BL against the larvae from 10th August had a decrease of 28 ASVs (Additional file 17). The comparison between FL and juveniles without osculum (JNO) showed 88 ASVs newly acquired, or with significantly increased relative abundances, in settled juveniles prior to osculum formation, hypothetically as a result of starting the interaction with the substrate (Fig. 6, Additional file 17). Within these newly colonized ASVs, we found representatives of *Enterobacterales*, *Pseudomonadals* and *Rhodobacterales* (Additional file 7, Additional file 18). The transition from juveniles without osculum (JNO) to juveniles with a functional osculum (JO) presented a total of 180 DA ASVs (Additional file 8). Notably, this transition involved the resurgence of ASV00003 (*Gammaprot*.; *Enterobacterales*; *Vibrio*), ASV00005 and ASV00013 (both *Gammaprot*.; *Pseudomonadales*; *Halomonas*), which were initially detected solely in free-living larvae from August 10th. In JNO, ASV00001 (Ca. *Beroebacter blanensis*) constituted more than 75% of the average relative abundance (avgRA), whereas in JO, it accounted for less than 15% of it (Fig. 5H). This shift can be attributed to the incorporation of new ASVs associated with the onset of filter-feeding capacity, consequently reducing the relative abundance of the dominant symbiont. In this case, samples were also collected at two different times (19th and 31th August), with the latter sampling revealing some changes among the JO samples, such as a noteworthy decrease in ASV00011 and ASV00006 classified as *Legionellales*, and an increase of ASV00001 (Additional file 18). A comparison between ASVs found in juveniles with a functional osculum and the original adults collected in the field, revealed striking differences in the ASVs abundances, with 123 DA ASVs being more abundant in juveniles with osculum and 177 DA ASVs being more abundant in adults (Fig. 6, Additional file 9). These disparities were primarily driven by the greater prevalence of *Enterobacterales, Flavobacteriales* and *Rhodobacterales* in juveniles, while *Synechococcales,* unclassified *Alphaproteobacteria* and *Nitrosopumilales* displayed higher relative abundances in adults (Additional file 9, Additional file 18). Both stages exhibited substantial relative abundances of *Pseudomonadales*; however, these were represented by distinct ASVs. Summary on the comparisons between different times are found in Additional file 17 and Additional file 10, and details on the taxonomic classification of the DA ASVs for each comparison are found in Additional file 18.

## Discussion

Our results represent a first approach to the dynamics of the microbial community composition across the ontogenetic cycle of the sponge *Crambe crambe.* Before our study, few papers delved into the differences in the structure and composition of the sponge microbiome during a comprehensive array of ontogenetic stages in LMA sponges [30, 44, 46, 47] and even fewer in HMA sponges [45]. Our overall comparison of the microbiota across adult individuals, brooded larvae, free-living larvae, settled juveniles with and without osculum revealed marked differences among them. Those differences appear to reflect the relative importance of the vertically and horizontally transmitted symbiotic complements at each stage.

### Bacterial composition of *Crambe crambe*

Like all demosponges, *C. crambe* has close associations with microbial communities [67]. This species has been categorized as an LMA sponge using electron microscopy and 16S rRNA gene amplicon sequencing [31]. We found that their most abundant microbial phyla across stages, including *Proteobacteria* (with *Gammaproteobacteria* being the most abundant class), *Bacteroidota* and *Cyanobacteria*, were recognized as consistent microbiome communities in this species, similar to what has previously been found in other LMA sponges [78] as well as in *C. crambe* [48, 79]. Furthermore, the presence of a specific ASV (Ca. *Beroebacter blanensis*), exhibiting varying relative abundances throughout different developmental stages, aligns with the previously documented dominance of a single symbiont within this species [48, 49, 79]. This symbiont is member of the newly described order of sponge symbionts, Ca. *Tethybacterales*. Members of this group share heterotrophic lifestyle and present differential abilities to use carbon, nitrogen and sulfur sources [49]. Few members of this bacterial group have been observed via microscopy, including the *C. crambe* symbiont [48], and determined to be vertically transmitted from the adult sponge to the embryo in the case of *Amphimedon queenslandica* [30]*, Tethya rubra* [80] and *Tedania* sp [47]. Moreover, members of Ca. *Tethybacterales* were generally present at really low abundances in seawater and sediment samples [49]. Although the morphology of Ca. *Beroebacter blanensis* has not been properly described yet, we observed a single symbiont morphotype maintained across ontogenetic stages, which was also previously observed by other authors [48, 62], and could potentially represent the main symbiont of *Crambe crambe*, Ca. *Beroebacter blanensis.*

Globally, adults and juveniles with osculum presented the highest diversity, while brooded larval and free-living larval stages showed much lower diversity, as observed during the life cycle of *Tedania* sp. [47]. This contrasts with the increase in diversity experienced during embryogenesis in the oviparous sponge *Ianthella basta*, which was potentially linked to the mechanism of vertical symbiont transmission that mostly occurs after oogenesis [44].

We have now compelling evidence that the structure and composition of the microbiome of *C. crambe* varies importantly through its ontogeny. Likely, some microbial classes are expected to play a role in how the sponge individual functions and interacts with the surrounding environment [15, 81]. Therefore, the differences in the microbiome found in the pre-competent larvae, free-living larvae and juveniles are indicative of the ability of the sponge to respond to changes in its environment and its internal milieu across its life cycle, demonstrating plasticity in their host-symbiont recognition systems. According to the metagenomic study of the main *C. crambe* symbiont, Ca. *B. blanensis* is able to use taurine as a source of carbon for growth [49]. Moreover, it is able to reduce sulphite to sulphide via assimilatory sulphite reductase. It encodes ABC transporters for the uptake of glycine betaine, a solute accumulated in marine phytoplankton, which can be used to maintain favourable osmotic tensions [82] and might serve as a carbon and nitrogen source in this symbiont. In support of this hypothesis, encode enzymes for the degradation of glycine betaine are found in Ca. *B. blanensis* [49].

### Microbiome variation during the brooding period of *Crambe crambe*

The fidelity in the vertical transmission of symbionts in sponges, particularly in *C. crambe*, is currently considered to be weak, given that, on average, the composition of adults and their released larvae is quite different [27]. This pattern contrasts with the classical theory that predicts that when microbes provide beneficial services to animals, these services should be faithfully transmitted. In fact, a recent comparative study on a wide array of animals found that the removal of vertically transmitted microbial symbionts resulted in a large reduction in host fitness [83]. Our study reveals that vertical transmission fidelity in *C. crambe* appears to be potentially primarily limited to its single dominant symbiont, which consistently persists throughout the entire ontogenetic cycle. However, additional microscopy and molecular techniques should be used to validate this finding. It is worth noting that the number of vertically transmitted symbionts could be more extensive, which is evidenced by the high similarity in composition between brooded larvae and adult individuals, which share up to 29 core ASVs (87% RA in the BL) belonging to 9 distinct bacterial classes (Fig. 5B, C). This finding suggests the potential significance of these ASVs in the developmental process of *C. crambe*, which may play some role during the larval stage. These putative functions include the ability to use different carbon sources, as demonstrated for Ca. *Berobacter blanensis* [49] or the production of arginine clues for larval settlement [84]. Regarding changes in relative abundance of ASVs, there were 96 ASVs that decreased in abundance or were not detected in larvae and 20 that increased, although these numbers might be considered with caution due to the limited number of adult biological replicates (i.e. 3 adults). This suggests that the bulk of the community composition may be transferred during the brooding process, although there is still some stochasticity in the process.

Moreover, our analysis revealed a higher degree of microbiome similarity between parental adults and their respective larvae, suggesting strong association and relative fidelity in the transmission of microbial taxa at the individual level. Notably, the majority of ASVs present in brooded larvae were also found in the adult microbiome, supporting the idea of vertical transmission from parents to offspring (Additional file 1). Furthermore, our results showed that brooded larvae possessed few ASVs not detected in their parents, albeit in a low proportion in terms of relative abundance (< 3%, Additional file 2). Among these exclusive ASVs found in brooded larvae, we identified taxa that are typically associated with human-related sources, including *Streptococcus*, *Acinetobacter*, *Pseudomonas*, and *Staphylococcus*. It is worth considering that the brooded larvae underwent a higher degree of manipulation under the binocular microscope, which was necessary to remove any remnants of adult tissue. This manipulation increases the likelihood of contamination from external sources, potentially explaining the presence of human-associated bacteria. Additionally, the lower microbial diversity observed in brooded larvae makes them more susceptible to the amplification of bacteria from other sources.

### Microbiome variation during the spawning period in *Crambe crambe*

Most sponge larvae are anchiplanic, being able to be in the seawater column for a brief period of time, between minutes and a few days [40]. During the free-swimming period, sponge larvae are usually not able to ingest bacteria from the seawater column (since they are lecithotrophic), but their epithelium is known to assimilate dissolved organic matter [85, 86]. However, an intriguing report on the feeding capacities of the crawling larva of *Halichondria panicea* was published in the 1990s [87], describing the ability of these sponge larvae to feed on both external and internal symbiotic bacteria. There is evidence of differential phagocytosis of internal symbionts during oogenesis [28, 38, 39, 61, 88, 89], embryogenesis [90], and larval metamorphosis [30], and even symbiont digestion in adult stages has been commonly documented [2, 91, 92], feeding on certain symbionts and therefore allowing other taxa to take up the niche and grow. In our study, it was only when larvae were released into the seawater that their microbial composition underwent a substantial shift, with changes in 101 ASVs and keeping a single dominant symbiont shared between brooded and free-swimming larvae (Fig. 5D). Unfortunately, the parents of the FL were unknown, which might also affect the observed changes in the microbiome community of larvae. However, their microbial communities showed more similarity to the microbial communities of the BL (from known parents) than to adult samples, suggesting that ontogenetic stage is the main driver of community composition in this species. The FL larvae from 9th August, collected the same day of the adult samples in the nearby water, had a more similar community to the brooded larvae than the FL larvae collected the day after. Most likely, the first ones were recently released from the same adults collected or close living related adults, making their community more similar to the BL. In turn, FL larvae from the day after could be larvae swimming in the water column for longer times and from further populations, given the complex local oceanographic features, and therefore, the community might have already been subjected to changes from the environment (different seawater microbiome) or even selective digestion within the larvae for nutrition. The FL became dominated by *Gammaproteobacteria* but depleted in other taxa, such as *Cyanobacteria*, *Alphaproteobacteria* and certain *Planctomycetes*. The reduction in the relative abundance of *Oxyphotobacteria*, which are involved in oxygenic photosynthesis [93], usually indicates a decrease in the carbon supply to the host [94], which is linked to deceleration in growth [95]. During the ontogeny of *Tedania* sp., the major shift is found in post-competent larvae at the free-swimming stage (8 h after release), with larger abundances of *Clostridium* sp. and *Bacteroides* than in previous and later stages [47].

An elegant study by Fieth and collaborators [30] proved the existence of metabolic complementation between sponge larvae and their microbial symbionts, with the bacteria providing arginine clues for settlement [84]. It could be that in *C. crambe* larvae, the community shifts occurring at the free-swimming stage are also involved in the metabolic pathways of settlement induction. In particular, the escalating abundances of *Firmicutes* (especially *Bacilli*) during the BL and FL stages, which also occur in the 48 h juveniles of *A. queenslandica*, could be somehow related to settlement in *C. crambe*. Although the exact function of this bacterial phylum within the sponge microbiome is unknown, an increase in the number of *Firmicutes* has been linked to stress in corals [96].

### Microbiome variation after settlement of *Crambe crambe* larvae

After settling and undergoing metamorphosis for approximately one week, the larvae of *C. crambe* transform into juveniles [63]. From a free-living larva to a settled juvenile still lacking an osculum, there was an increase (or acquisition) in a total of 88 ASVs, and no ASVs were detected to reduce their relative abundance. After settling into benthic habitats, it is hypothesized here that microbes from the surface of rocks can enter juvenile tissue. This explanation stems from the observation of penetration of microbes from seawater across sponge epithelia, which has been documented in some adult sponges [97]. Before the osculum is formed, it is thought that the settled juvenile relies mostly on its vitelline reserves [85] and phagocytosis of symbiotic bacteria (discussed above). In *Amphimedon queenslandica*, Fieth et al. [30] showed faithful vertical transmission patterns from adults and brooded larvae to free-swimming larvae. However, the major shift in the communities occurred in the first 48 h after larval settlement and prior to osculum formation, when taxa such as *Actinobacteria*, *Bacteroidetes*, and *Firmicutes*, previously undetectable, become dominant in the communities. This shift in the microbial composition is attributed to the ingestion of the predominant symbiont, coupled with the influence of environmentally derived microbes originating from either the benthic settlement substrate or the surrounding seawater [30]. Unfortunately, we could not assess the microbial composition of the substrate or the surrounding seawater to evaluate their potential effect on shaping the microbiome of the developing juveniles.

Upon the formation of the osculum, *C. crambe* acquires filter-feeding capacity, resulting in a large influx of environmental microbes, which coincides with a remarkable increase in microbial diversity observed between settled larvae without osculum and the juvenile phase with osculum. Up to 120 ASVs were newly acquired or increased their abundances in the transition to this new life stage, while 60 ASVs from the previous stage were lost or drastically reduced their abundances. Notably, juveniles without an osculum (JNO) and juveniles with a functional osculum (JO) share up to 12 ASVs from 3 bacterial classes (~ 75% avgRA in JNO and ~ 20% avgRA in JO). These ASVs are probably important members of the community that were never digested or expelled and that have outcompeted the invasion of environmental microbes.

The juvenile phase exhibits diversity values that are similar to those found in adult sponges, suggesting the progressive development of a more complex microbial community within the sponge. However, drastic changes in microbial abundances still need to occur during the transition from the juvenile to the adult stage, since over 300 differentially abundant ASVs were detected. These data indicate that the juvenile stage requires additional time to selectively acquire and curate specific bacterial taxa, ultimately leading to the establishment of the complex, host specific, microbial community in the adult. However, we need to acknowledge the fact that experimental conditions might limit the access to the real seawater microbiome, hampering the complete restoration of the adult microbiome. The process of microbial community maturation in *C. crambe* highlights the dynamic nature of the symbiotic relationships.

## Conclusions

The findings from this study underscore a delicate intertwining between ontogeny and microbiome composition in the sponge *C. crambe*, with distinct symbionts in each stage. Our results suggest that the dominant symbiont, Candidatus *Beroebacter blanensis*, may be vertically transmitted. We hypothesize that such a transmission has been selected through evolution because this bacterium may provide some compounds crucial to the non-feeding (i.e., lecithotrophic) developmental stages of the sponge, where it constitutes over 70% of the microbiome's relative abundance. The unique microbial compositions observed in each ontogenetic phase likely arise from a combination of vertical and horizontal transmission processes, wherein the adult microbiome and the environmental microbiome (from substrate and seawater) are incorporated into the larval stages. A notable shift in the composition of the microbial community occurs as the juveniles acquire filtering capacity upon the formation of the osculum, facilitating the microbiome composition of juveniles to progressively resemble that of adult individuals. It is important for future research to examine the potential metabolic contributions of these specific symbionts in each ontogenetic phase. Investigating whether they provide nutritional resources, settlement cues, or others will enhance our understanding of symbiotic interactions and their contribution to the developmental processes of *C. crambe* and other sponges, both LMA and HMA. Additionally, investigating the mechanisms underlying the vertical transmission of the dominant symbiont and the factors shaping the acquisition of other symbionts during larval stages would provide valuable insights into the establishment and dynamics of the sponge microbiome.

### Supplementary Information

**Additional file 1: Figure S1.** Number of shared ASVs between adult individuals and their own brooded larvae from the three individuals analysed. The Upset plot shows inclusive intersections between analysed pairs. Pairs originating from the same individual are marked in the same colour in the matrix. Set size corresponds to core community values (present in all the replicates from the same individual) and bars represent the size (num. ASVs) of the indicated interaction in the matrix ordered by decreasing values. Venn diagrams show specific comparisons between adults-brooded larvae pairs from the same individual.

**Additional file 2: Figure S2**. Barplot showing the average relative abundance of the exclusive ASVs (shown in numbers on top of the bars) in each ontogenetic stage. Taxonomic composition is shown at class level.

**Additional file 3: Figure S3.** Upset plot showing inclusive intersections between *C.crambe* consecutive stages analysed, including different times for FL and JO stages. When the different times are considered they are shown as FL1 (09 Aug), FL2 (10 Aug) and JO1 (19 Aug) and JO2 (31 Aug). Set size corresponds to core community values (assessed at 70% of replicates) and bars represent the size (no. ASVs) of the indicated interaction in the matrix ordered by decreasing values.

**Additional file 4: Figure S4.** Shared taxa at order level between ontogenetic stages of *C.crambe*. **A.** Table showing core communities calculated based on the number of microbial orders found in 100% and 70% of the total number of replicates in each stage (columns 4 and 5, respectively). Percentages indicate the average relative abundance of the core ASVs in each stage. **B.** Barplot showing the relative abundance of the 10 shared microbial orders across all stages. **C.** Venn diagram of the shared orders (at 70% of replicates) between developmental stages.

**Additional file 5: Figure S5.** Bubble plots representing the relative abundances (sqrt transformed) of Differentially Abundant (DA) ASVs between Adult and Brooding Larvae. For representative purposes, we only show the 100 most abundant ASVs (when present) in each comparison.

**Additional file 6: Figure S6.** Bubble plots representing the relative abundances (sqrt transformed) of Differentially Abundant (DA) ASVs between Brooding Larvae and Larvae Free Living. For representative purposes, we only show the 100 most abundant ASVs (when present) in each comparison.

**Additional file 7: Figure S7.** Bubble plots representing the relative abundances (sqrt transformed) of Differentially Abundant (DA) ASVs between Larave Free Living and Juvenile with No Osculum. For representative purposes, we only show the 100 most abundant ASVs (when present) in each comparison.

**Additional file 8: Figure S8.** Bubble plots representing the relative abundances (sqrt transformed) of Differentially Abundant (DA) ASVs between Juvenile with No Osculum and Juvenile with Osculum. For representative purposes, we only show the 100 most abundant ASVs (when present) in each comparison.

**Additional file 9: Figure S9.** Bubble plots representing the relative abundances (sqrt transformed) of Differentially Abundant (DA) ASVs between Juvenile with Osculum and Adult. For representative purposes, we only show the 100 most abundant ASVs (when present) in each comparison.

**Additional file 10: Figure 10.** Differentially abundant (DA) ASVs across the reproductive cycle of the sponge *C. crambe,* with comparisons for the different sampling times. Values represent the number of ASVs identified at higher/lower relative abundances in the comparison between the consecutive ontogeny phases. Abundances and taxonomy of DA ASVs for each comparison can be found in Supplementary Figs. 5–9 and Additional file 18: Table S7.

**Additional file 11**: Results of the statistical tests performed for **A**. Alpha diversity metrics (Anova and pair-wise comparisons) for the whole dataset, **B**. Beta diversity (Permutest and Permanova), **C**. Adults and brooded larvae comparisons (Bray–Curtis and Jaccard similarity) and **D.** Abundance of ASV0001 across different stages.

**Additional file 12: Table S1.** Taxonomic composition at Phylum level for each ontogenetic stage of *C. Crambe* ordered in descending total abundance (last column). Numbers represent percentages (%) of average Relative abundances. Taxa with relative abundances < 0.01% are grouped as “Others”. AD: Adult, BL: Brooding Larvae, FL: Free Living larvae, JNO: Juvenile No Osculum, JO: Juvenile with Osculum, Total: mean relative abundance across all samples.

**Additional file 13: Table S2.** Taxonomic composition at Class level for each ontogenetic stage of *C. crambe* ordered in descending total abundance (last column). Numbers represent percentages (%) of average Relative abundances. Taxa with relative abundances < 0.01% are grouped as “Others”. AD: Adult, BL: Brooding Larvae, FL: Free Living larvae, JNO: Juvenile No Osculum, JO: Juvenile with Osculum, Total: mean relative abundance across all samples. In Taxonomy column, A: Archaea, B: Bacteria.

**Additional file 14: Table S3.** Shannon diversity and InvSimpson index for each sample. Information on Individual, Replicate, Stage and Sampling date are also shown as separated columns.

**Additional file 15:Table S4**. Microbial core communities of each developmental stage of *C. crambe.* Core communities are calculated based on the number of ASVs found in 100% and 70% of the total number of replicates in each stage (columns 4 and 5, respectively). Percentages indicate the average relative abundance of the core ASVs in each stage. In grey, the values used for comparisons in the Upset plot.

**Additional file 16: Table S5.** Abundances and taxonomic identification (Class level) of the shared ASVs between consecutive ontogenetic stages (shown in Additional file 15: Table S4). In yellow, ASVs that were found at significant difference Abundances (see Additional file 18: Table S7).

**Additional file 17: Table S6.** Differential Abundance (DA) analysis between the different ontogenetic stages for *C. crambe.* In grey we show the comparisons that appear in the Fig. 6. In the case of Free-Living larvae (FL) and Juveniles with Osculum (JO), when the different times are considered they are shown as FL1 (09 Aug), FL2 (10 Aug) and JO1 (19 Aug) and JO2 (31 Aug).

**Additional file 18: Table S7.** Abundances and taxonomic identification of the Differentially Abundant (DA) ASVs detected in Additional file 17: Table S6. Each comparison is found in a separate sheet.

## Data Availability

Raw sequence data are available from the NCBI SRA under project PRJNA990364 (https://www.ncbi.nlm.nih.gov/sra/?term=PRJNA990364) and metadata information is found in Additional file 14.

## Abbreviations

ASV: Amplicon sequence variant
MAG: Metagenome-assembled-genome
AvgRA: Average relative abundance
HMA: High microbial abundance
LMA: Low microbial abundance
AD: Adult
BL: Brooded pre-competent larva
FL: Free-living larvae
JNO: Juvenile with no osculum
JO: Juvenile with osculum
