# Supplementary material for: Microbiome changes through the ontogeny of the marine sponge Crambe crambe
Source: Environ Microbiome. 2024 Mar 11;19:15. doi: 10.1186/s40793-024-00556-7 (PMC10929144; doi:10.1186/s40793-024-00556-7)
Supplement: Supplementary file 1 — Additional file 1: Figure S1. Number of shared ASVs between adult individuals and their own brooded larvae from the three individuals analysed. The Upset plot shows inclusive intersections between analysed pairs. Pairs originating from the same individual are marked in the same colour in the matrix. Set size corresponds to core community values (present in all the replicates from the same individual) and bars represent the size (num. ASVs) of the indicated interaction in the matrix ordered by decreasing values. Venn diagrams show specific comparisons between adults-brooded larvae pairs from the same individual. [file 40793_2024_556_MOESM1_ESM.pdf]

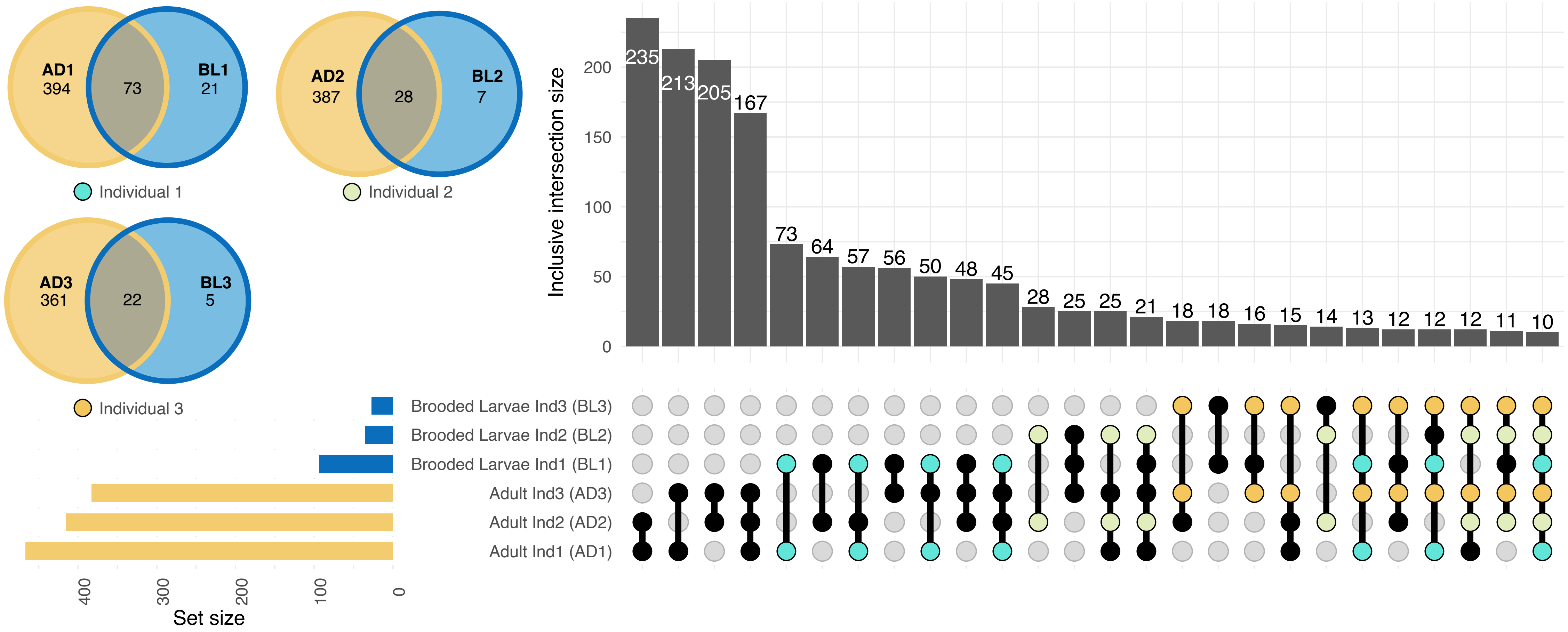

AD1
394
73
BL1
21
Individual 1
AD2
387
28
BL2
7
Individual 2
AD3
361
22
BL3
5
Individual 3
Inclusive intersection size
0
50
100
150
200
235
213
205
167
73
64
57
56
50
48
45
28
25
25
21
18
18
16
15
14
13
12
12
12
11
10
Brooded Larvae Ind3 (BL3)
Brooded Larvae Ind2 (BL2)
Brooded Larvae Ind1 (BL1)
Adult Ind3 (AD3)
Adult Ind2 (AD2)
Adult Ind1 (AD1)
400
300
200
100
0
Set size
