# Supplementary material for: Microbiome changes through the ontogeny of the marine sponge Crambe crambe
Source: Environ Microbiome. 2024 Mar 11;19:15. doi: 10.1186/s40793-024-00556-7 (PMC10929144; doi:10.1186/s40793-024-00556-7)
Supplement: Supplementary file 2 — Additional file 2: Figure S2. Barplot showing the average relative abundance of the exclusive ASVs (shown in numbers on top of the bars) in each ontogenetic stage. Taxonomic composition is shown at class level. [file 40793_2024_556_MOESM2_ESM.pdf]

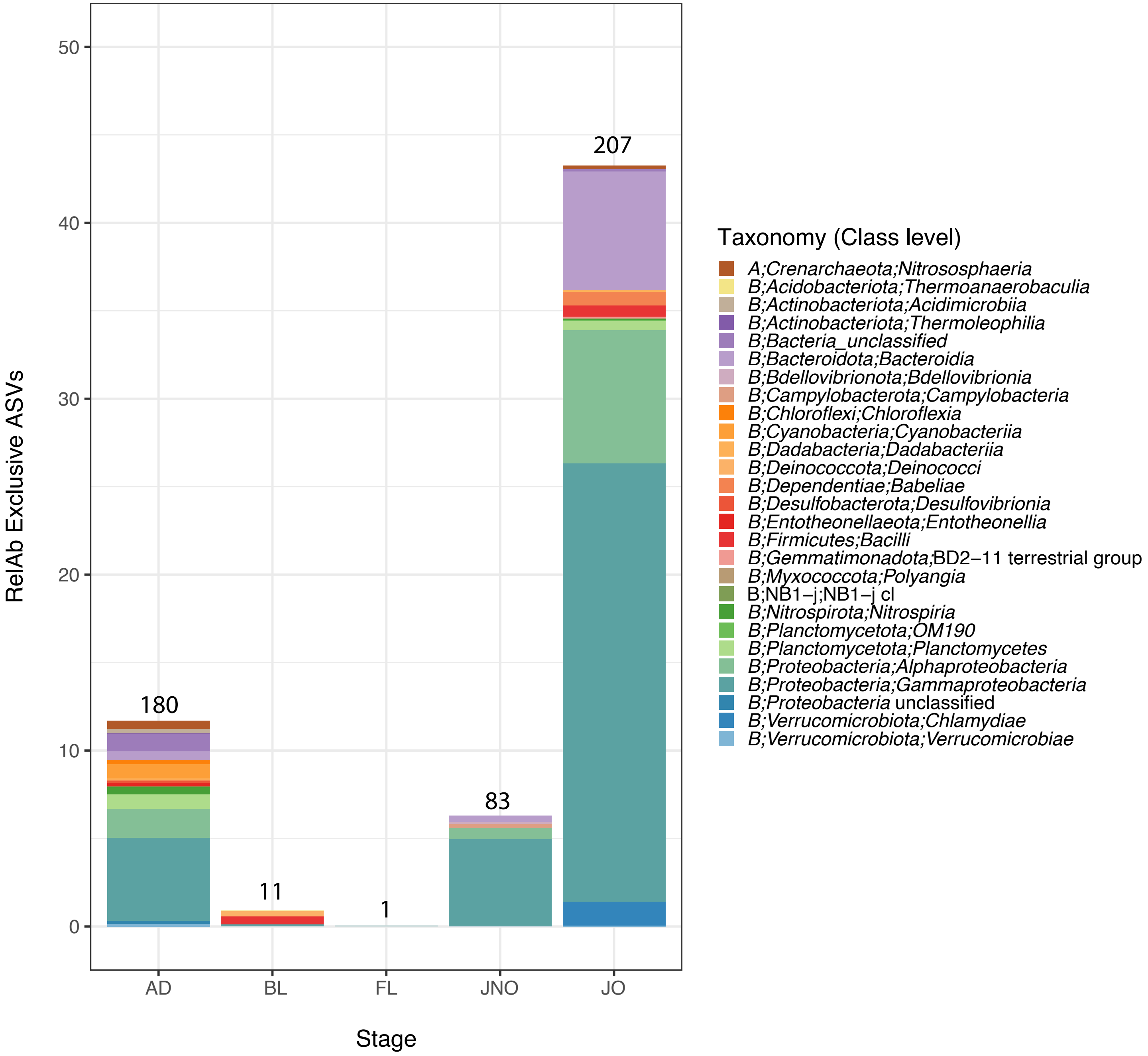

RelAb Exclusive ASVs
0
10
20
30
40
50
180
11
1
83
207
AD
BL
FL
JNO
JO
Stage
Taxonomy (Class level)
A;Crenarchaeota;Nitrososphaeria
B;Acidobacteriota;Thermoanaerobaculia
B;Actinobacteriota;Acidimicrobiia
B;Actinobacteriota;Thermoleophilia
B;Bacteria_unclassified
B;Bacteroidota;Bacteroidia
B;Bdellovibrionota;Bdellovibrionia
B;Campylobacterota;Campylobacteria
B;Chloroflexi;Chloroflexia
B;Cyanobacteria;Cyanobacteriia
B;Dadabacteria;Dadabacteriia
B;Deinococcota;Deinococci
B;Dependentiae;Babeliae
B;Desulfobacterota;Desulfovibrionia
B;Entotheonellaeota;Entotheonellia
B;Firmicutes;Bacilli
B;Gemmatimonadota;BD2−11 terrestrial group
B;Myxococcota;Polyangia
B;NB1−j;NB1−j cl
B;Nitrospirota;Nitrospiria
B;Planctomycetota;OM190
B;Planctomycetota;Planctomycetes
B;Proteobacteria;Alphaproteobacteria
B;Proteobacteria;Gammaproteobacteria
B;Proteobacteria unclassified
B;Verrucomicrobiota;Chlamydiae
B;Verrucomicrobiota;Verrucomicrobiae
