# Supplementary material for: Microbiome changes through the ontogeny of the marine sponge Crambe crambe
Source: Environ Microbiome. 2024 Mar 11;19:15. doi: 10.1186/s40793-024-00556-7 (PMC10929144; doi:10.1186/s40793-024-00556-7)
Supplement: Supplementary file 3 — Additional file 3: Figure S3. Upset plot showing inclusive intersections between C.crambe consecutive stages analysed, including different times for FL and JO stages. When the different times are considered they are shown as FL1 (09 Aug), FL2 (10 Aug) and JO1 (19 Aug) and JO2 (31 Aug). Set size corresponds to core community values (assessed at 70% of replicates) and bars represent the size (no. ASVs) of the indicated interaction in the matrix ordered by decreasing values. [file 40793_2024_556_MOESM3_ESM.pdf]

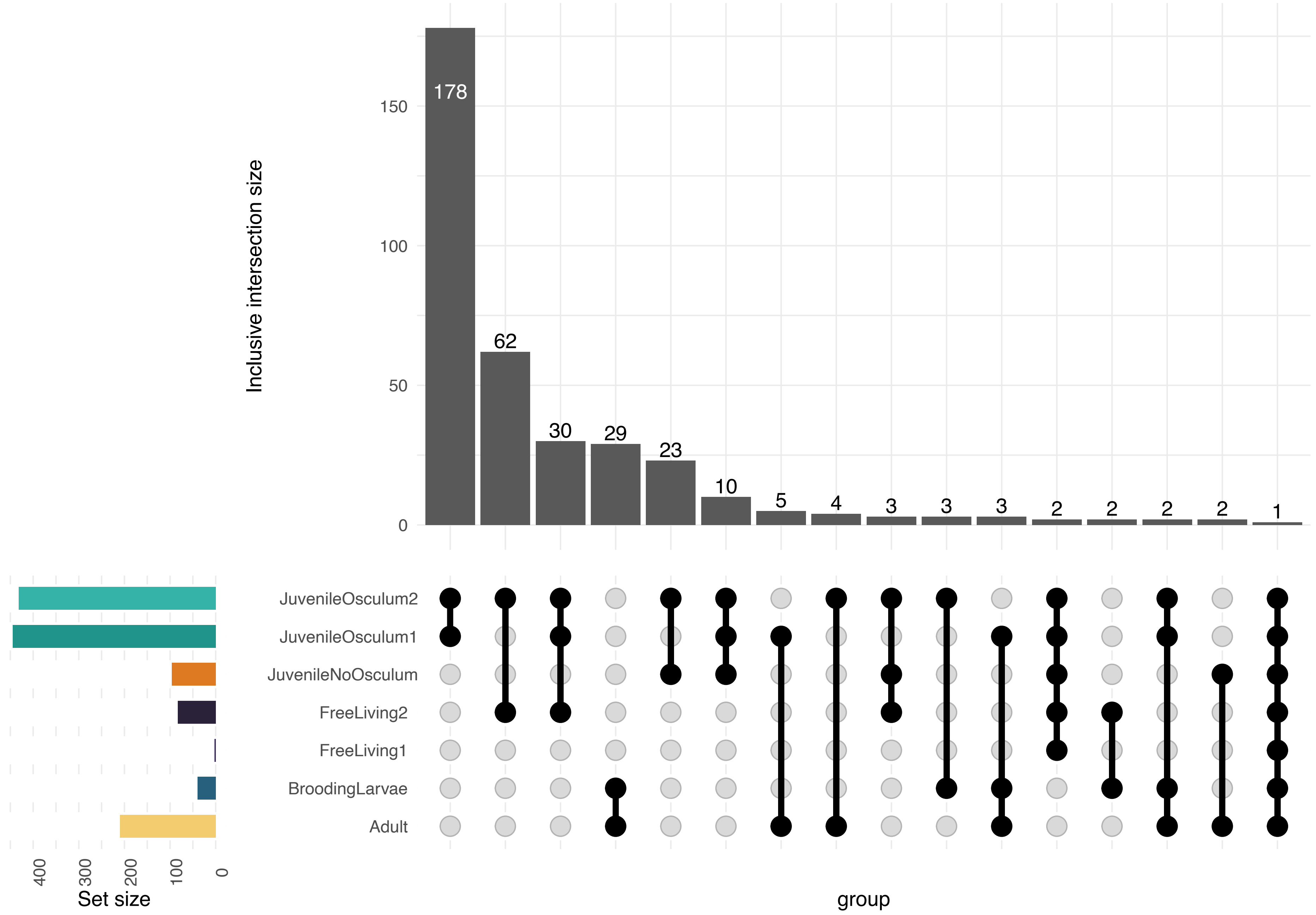
178
62
30
29
23
10
5
4
3
3
3
2
2
2
2
1
0
50
100
150
Inclusive intersection size
JuvenileOsculum2
JuvenileOsculum1
JuvenileNoOsculum
FreeLiving2
FreeLiving1
BroodingLarvae
Adult
400
300
200
100
0
Set size
group
