# Supplementary material for: Microbiome changes through the ontogeny of the marine sponge Crambe crambe
Source: Environ Microbiome. 2024 Mar 11;19:15. doi: 10.1186/s40793-024-00556-7 (PMC10929144; doi:10.1186/s40793-024-00556-7)

A.

Stage	No. replicates	Total taxa	CORE 100% (Freq. / %relab)	CORE 70% (Freq. / %relab)
Adult (AD)	9	233	70/ 98.9%	95/ 99.6%
Brooding Larvae (BL)	13	226	26 / 95.5%	55 / 98.2%
Free Living (FL)	10	124	9 / 95.2%	20 / 97.6%
Juvenile without osculum (JNO)	4	81	12/ 95.2%	25 / 99.5%
Juvenile with osculum (JO)	8	194	51 / 97.3%	90 / 99.3%

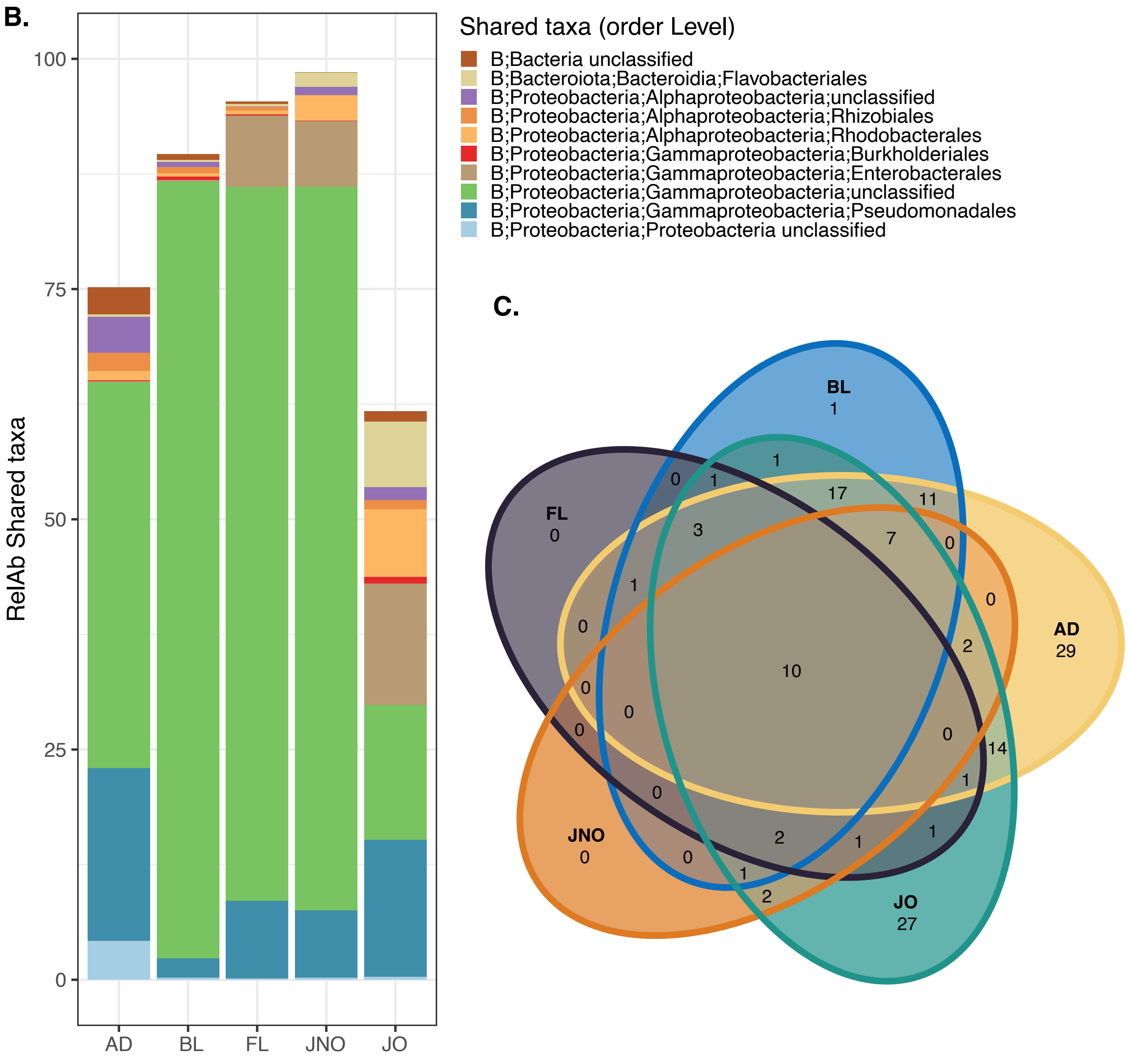

Supplement: Supplementary file 4 — Additional file 4: Figure S4. Shared taxa at order level between ontogenetic stages of C.crambe. A. Table showing core communities calculated based on the number of microbial orders found in 100% and 70% of the total number of replicates in each stage (columns 4 and 5, respectively). Percentages indicate the average relative abundance of the core ASVs in each stage. B. Barplot showing the relative abundance of the 10 shared microbial orders across all stages. C. Venn diagram of the shared orders (at 70% of replicates) between developmental stages. [file 40793_2024_556_MOESM4_ESM.pdf]
