# Supplementary figures and images for: Microbiome changes through the ontogeny of the marine sponge Crambe crambe
Source: Environ Microbiome. 2024 Mar 11;19:15. doi: 10.1186/s40793-024-00556-7 (PMC10929144; doi:10.1186/s40793-024-00556-7)

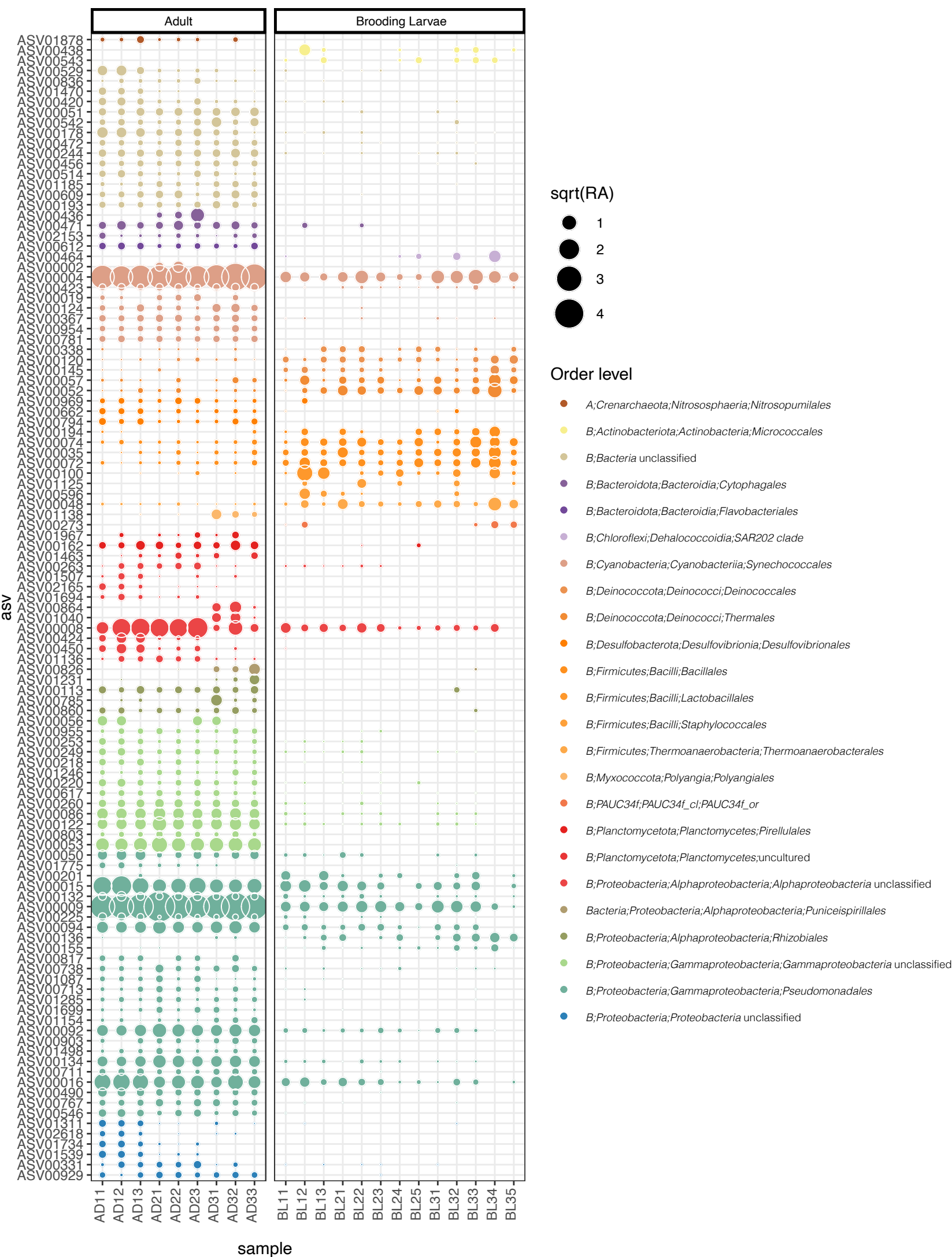

Supplement: Supplementary file 5 — Additional file 5: Figure S5. Bubble plots representing the relative abundances (sqrt transformed) of Differentially Abundant (DA) ASVs between Adult and Brooding Larvae. For representative purposes, we only show the 100 most abundant ASVs (when present) in each comparison. [file 40793_2024_556_MOESM5_ESM.pdf]

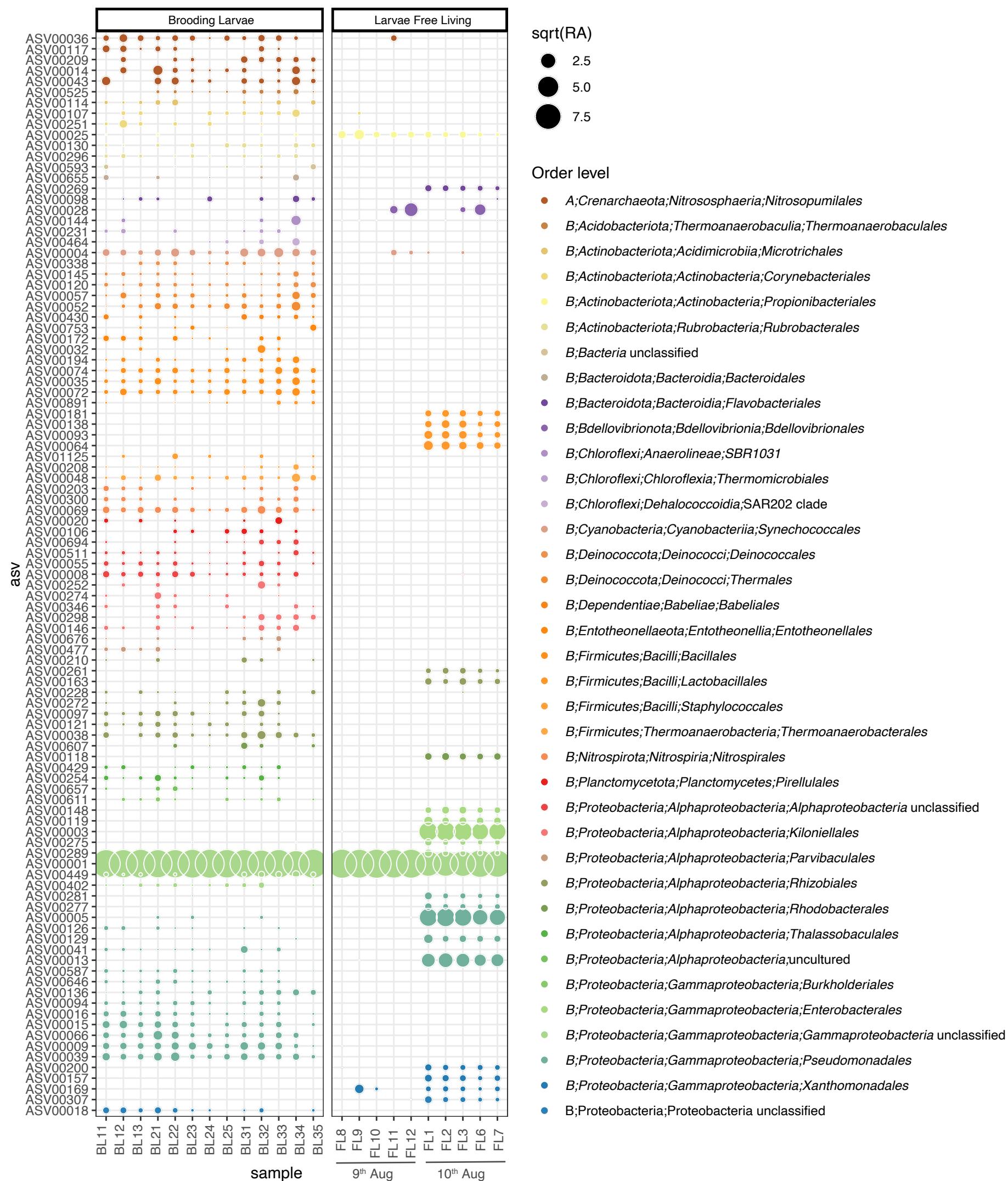

Supplement: Supplementary file 6 — Additional file 6: Figure S6. Bubble plots representing the relative abundances (sqrt transformed) of Differentially Abundant (DA) ASVs between Brooding Larvae and Larvae Free Living. For representative purposes, we only show the 100 most abundant ASVs (when present) in each comparison. [file 40793_2024_556_MOESM6_ESM.pdf]

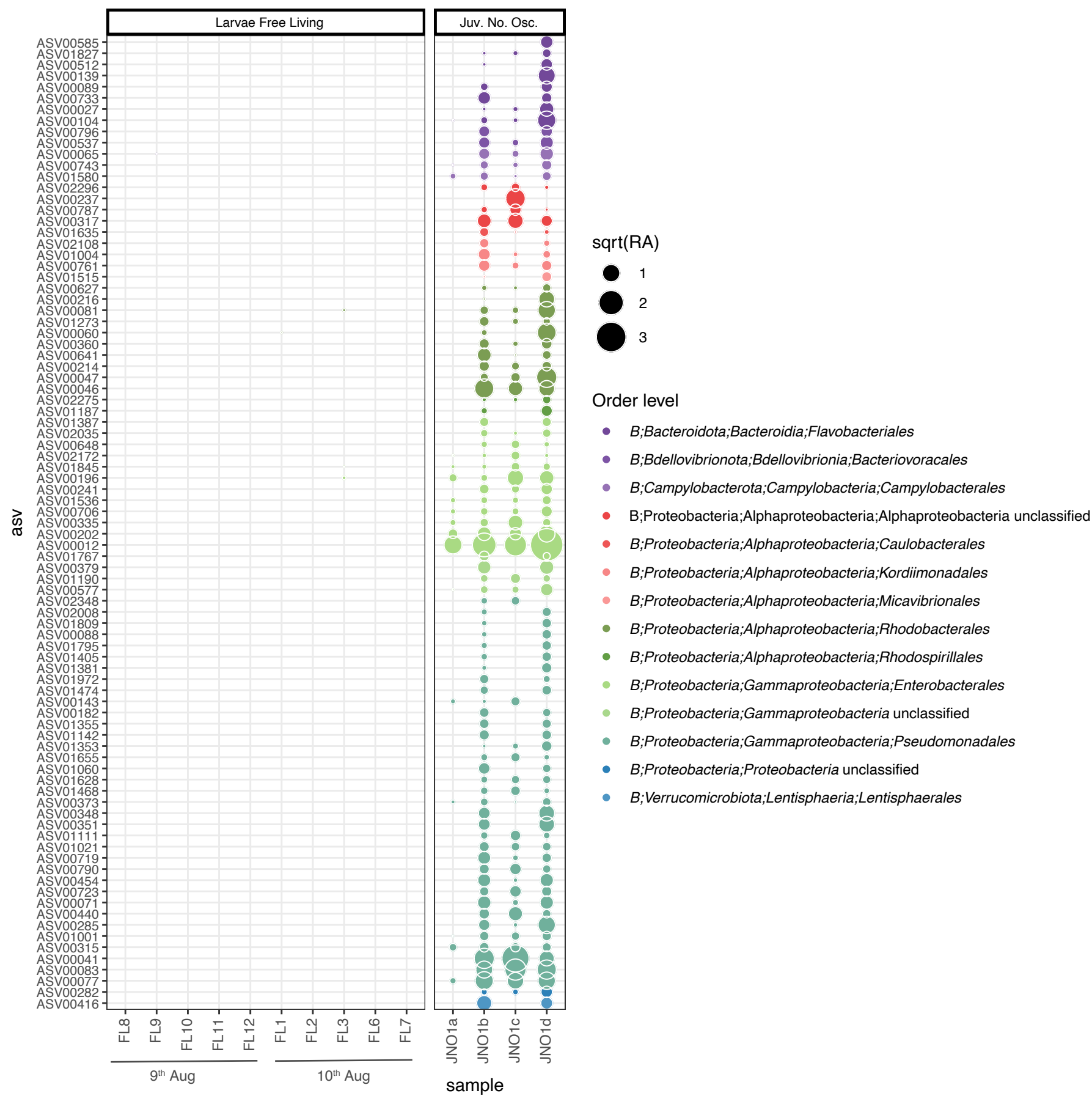

Supplement: Supplementary file 7 — Additional file 7: Figure S7. Bubble plots representing the relative abundances (sqrt transformed) of Differentially Abundant (DA) ASVs between Larave Free Living and Juvenile with No Osculum. For representative purposes, we only show the 100 most abundant ASVs (when present) in each comparison. [file 40793_2024_556_MOESM7_ESM.pdf]

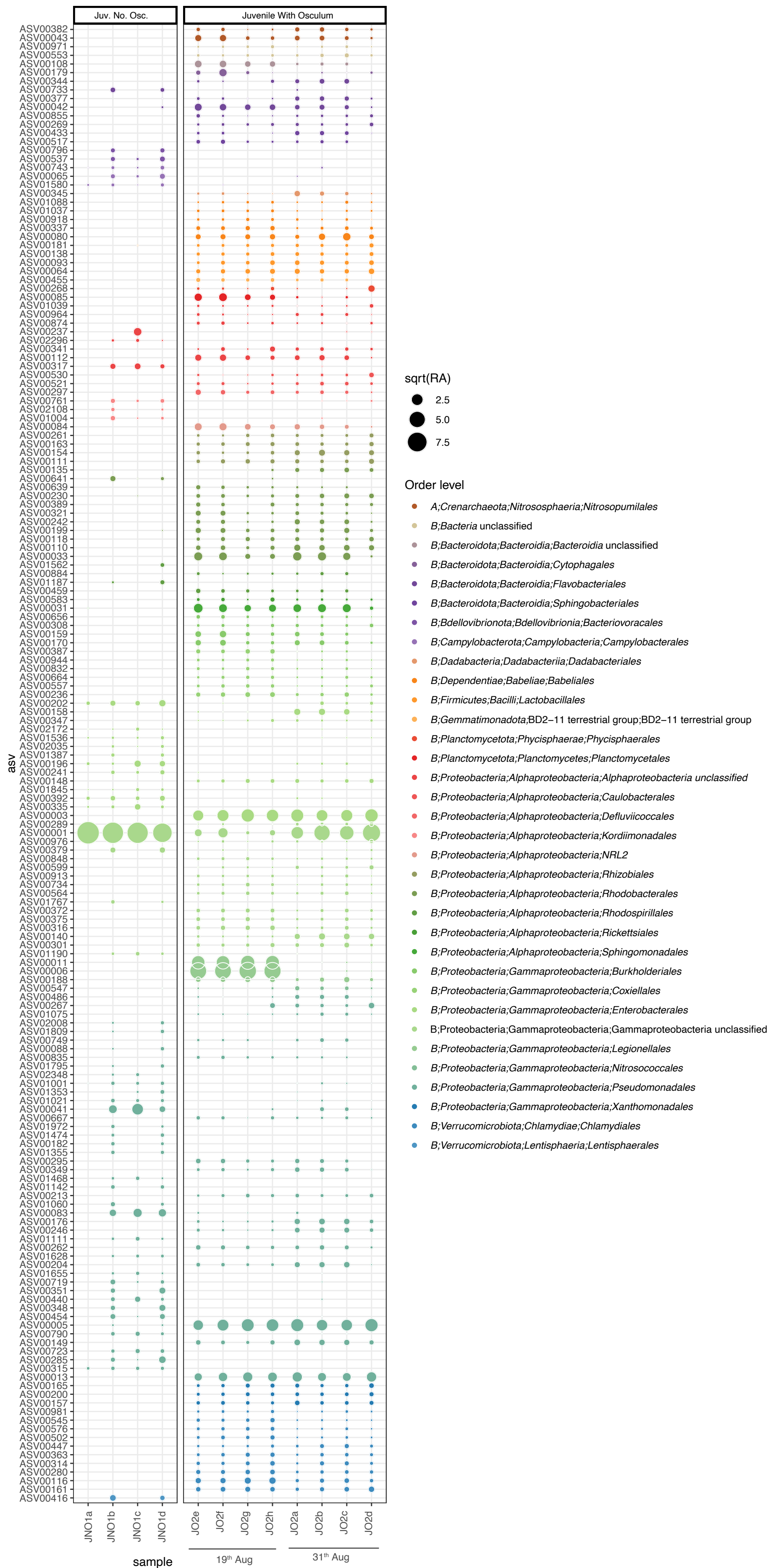

Supplement: Supplementary file 8 — Additional file 8: Figure S8. Bubble plots representing the relative abundances (sqrt transformed) of Differentially Abundant (DA) ASVs between Juvenile with No Osculum and Juvenile with Osculum. For representative purposes, we only show the 100 most abundant ASVs (when present) in each comparison. [file 40793_2024_556_MOESM8_ESM.pdf]

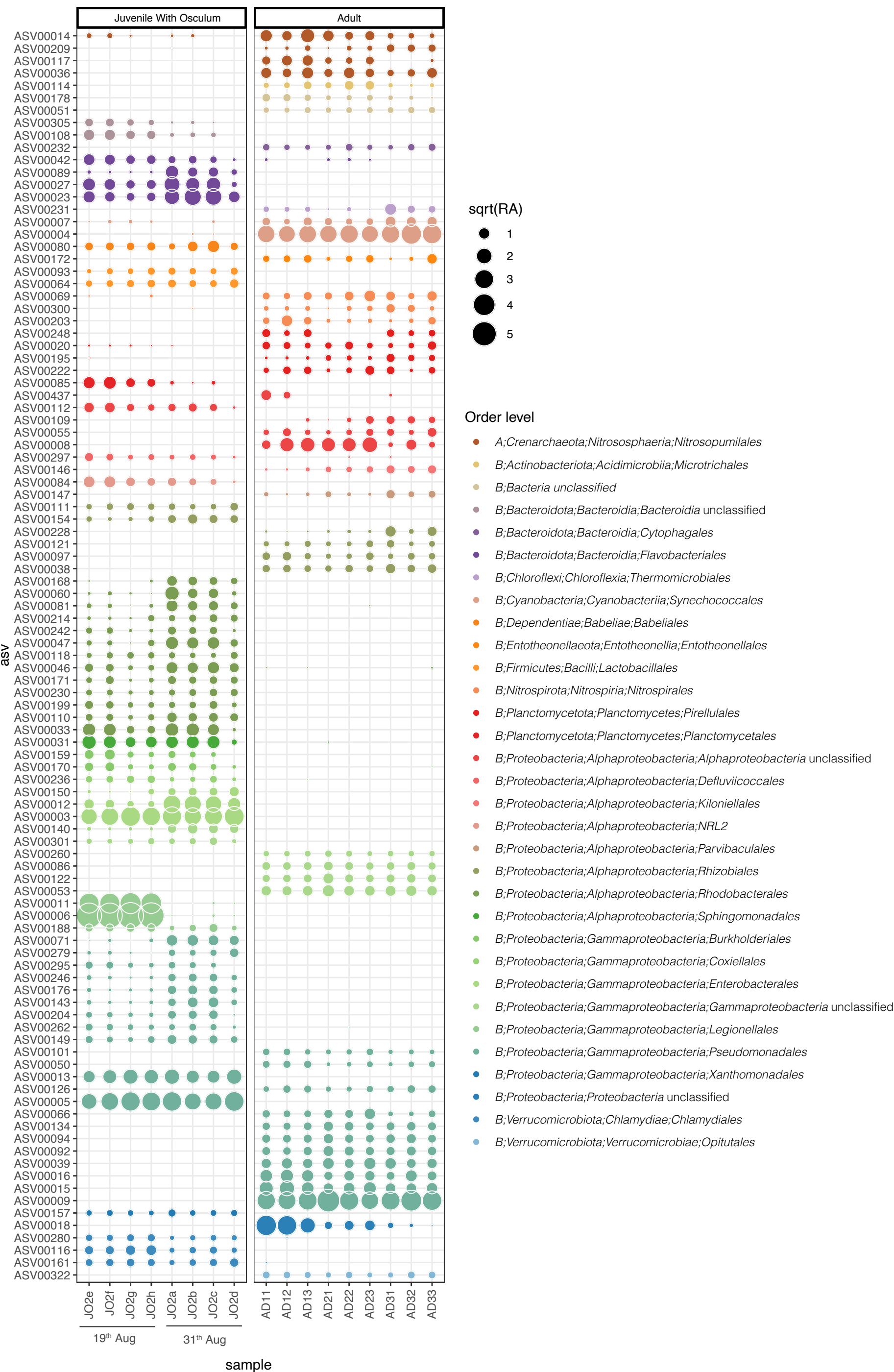

Supplement: Supplementary file 9 — Additional file 9: Figure S9. Bubble plots representing the relative abundances (sqrt transformed) of Differentially Abundant (DA) ASVs between Juvenile with Osculum and Adult. For representative purposes, we only show the 100 most abundant ASVs (when present) in each comparison. [file 40793_2024_556_MOESM9_ESM.pdf]

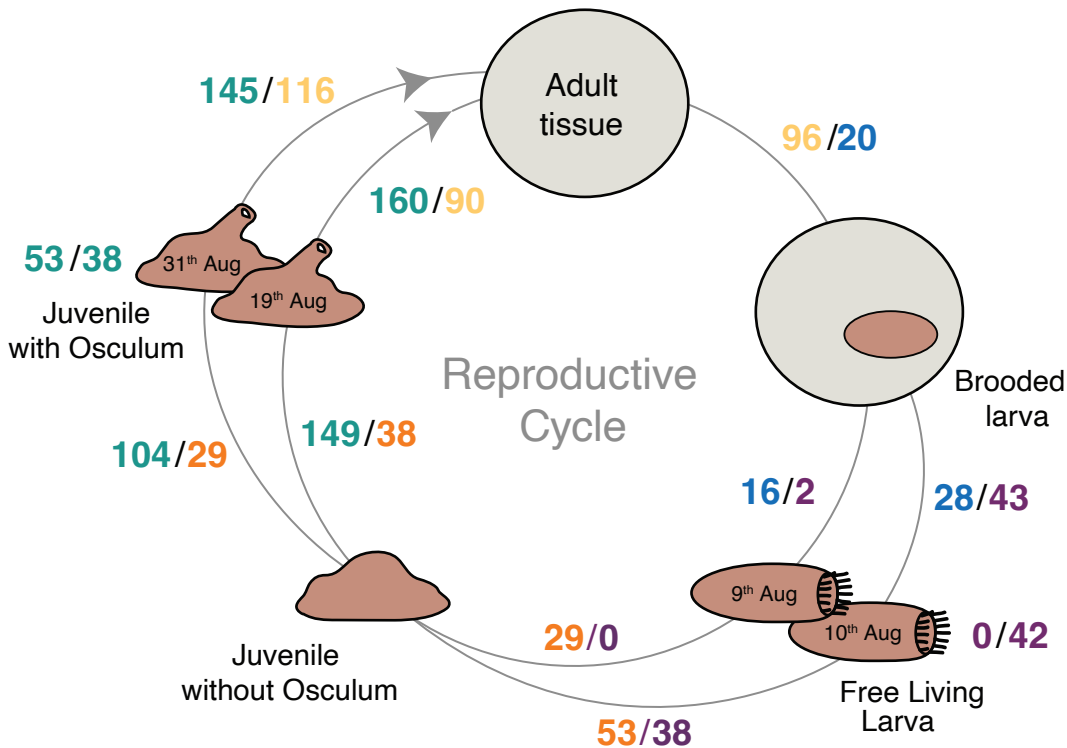

Supplement: Supplementary file 10 — Additional file 10: Figure 10. Differentially abundant (DA) ASVs across the reproductive cycle of the sponge C. crambe, with comparisons for the different sampling times. Values represent the number of ASVs identified at higher/lower relative abundances in the comparison between the consecutive ontogeny phases. Abundances and taxonomy of DA ASVs for each comparison can be found in Supplementary Figs. 5–9 and Additional file 18: Table S7. [file 40793_2024_556_MOESM10_ESM.pdf]
