# Supplementary material for: Microbiome changes through the ontogeny of the marine sponge Crambe crambe
Source: Environ Microbiome. 2024 Mar 11;19:15. doi: 10.1186/s40793-024-00556-7 (PMC10929144; doi:10.1186/s40793-024-00556-7)
Supplement: Supplementary file 11 — Additional file 11: Results of the statistical tests performed for A. Alpha diversity metrics (Anova and pair-wise comparisons) for the whole dataset, B. Beta diversity (Permutest and Permanova), C. Adults and brooded larvae comparisons (Bray–Curtis and Jaccard similarity) and D. Abundance of ASV0001 across different stages. [file 40793_2024_556_MOESM11_ESM.docx]

**Statistical analyses**

Results of the statistical tests performed for **A**. Alpha diversity metrics (Anova and pair-wise comparisons) for the whole dataset, **B**. Beta diversity (Permutest and Permanova), and **C**. Adults and brooded larvae comparisons (Bray-Curtis and Jaccard similarity).

**A. Alpha diversity**

**Shannon**

Anova for Shannon

Analysis of Variance Table

| Response: | shannonH | |  |  |  |
| --- | --- | --- | --- | --- | --- |
|  | Df | Sum Sq | Mean Sq | F value | Pr(>F) |
| Stage | 4 | 54.282 | 13.570 | 35.07 | **1.876e-11 ***** |
| Residuals | 33 | 12.769 | 0.387 |  |  |

---

Signif. codes: 0 ‘***’ 0.001 ‘**’ 0.01 ‘*’ 0.05 ‘.’ 0.1 ‘ ’ 1

Pair-wise comparisons

Tukey multiple comparisons of means

95% family-wise confidence level

Fit: aov(formula = shannonH ~ Stage, data = Infotable)

$Stage

|  | diff | lwr | upr | p adj |
| --- | --- | --- | --- | --- |
| BL-AD | -2.52 | -3.67 | -1.37 | **0.00** |
| FL-AD | -2.75 | -3.94 | -1.57 | **0.00** |
| JNO-AD | -2.49 | -3.86 | -1.12 | **0.00** |
| JO-AD | 0.03 | -1.18 | 1.25 | 1.00 |
| FL-BL | -0.23 | -0.99 | 0.52 | 0.90 |
| JNO-BL | 0.03 | -1.00 | 1.05 | 1.00 |
| JO-BL | 2.55 | 1.75 | 3.36 | **0.00** |
| JNO-FL | 0.26 | -0.80 | 1.32 | 0.95 |
| JO-FL | 2.79 | 1.93 | 3.64 | **0.00** |
| JO-JNO | 2.52 | 1.43 | 3.62 | **0.00** |

**InvSimpson**

Anova for InvSimpson

Analysis of Variance Table

| Response: | invSimp |  |  |  |  |
| --- | --- | --- | --- | --- | --- |
|  | Df | Sum Sq | Mean Sq | F value | Pr(>F) |
| Stage | 4 | 665.36 | 166.34 | 13.38 | **1.382e-06***** |
| Residuals | 33 | 410.06 | 12.426 |  |  |

---

Signif. codes: 0 ‘***’ 0.001 ‘**’ 0.01 ‘*’ 0.05 ‘.’ 0.1 ‘ ’ 1

Pair-wise comparisons

Tukey multiple comparisons of means

95% family-wise confidence level

Fit: aov(formula = invSimp ~ Stage, data = Infotable)

$Stage

|  | diff | lwr | upr | p adj |
| --- | --- | --- | --- | --- |
| BL-AD | -4.54 | -11.05 | 1.98 | 0.28 |
| FL-AD | -4.15 | -10.84 | 2.54 | 0.40 |
| JNO-AD | -4.17 | -11.93 | 3.60 | 0.54 |
| JO-AD | 5.95 | -0.93 | 12.84 | 0.12 |
| FL-BL | 0.39 | -3.89 | 4.66 | 1.00 |
| JNO-BL | 0.37 | -5.44 | 6.18 | 1.00 |
| JO-BL | 10.49 | 5.92 | 15.06 | **0.00** |
| JNO-FL | -0.02 | -6.03 | 6.00 | 1.00 |
| JO-FL | 10.10 | 5.28 | 14.92 | **0.00** |
| JO-JNO | 10.12 | 3.89 | 16.35 | **0.00** |

**Effect of time on Alpha diversity**

**Free-living larvae**

Anova for Shannon

Analysis of Variance Table

| Response: | invSimp |  |  |  |  |
| --- | --- | --- | --- | --- | --- |
|  | Df | Sum Sq | Mean Sq | F value | Pr(>F) |
| Date | 1 | 2.97 | 2.97 | 38.25 | **0.0002***** |
| Residuals | 8 | 0.62 | 0.06 |  |  |

---

Signif. codes: 0 ‘***’ 0.001 ‘**’ 0.01 ‘*’ 0.05 ‘.’ 0.1 ‘ ’ 1

Anova for InvSimp

Analysis of Variance Table

| Response: | invSimp |  |  |  |  |
| --- | --- | --- | --- | --- | --- |
|  | Df | Sum Sq | Mean Sq | F value | Pr(>F) |
| Date | 1 | 4.7 | 4.773 | 22.87 | **0.001**** |
| Residuals | 8 | 1.6 | 0.208 |  |  |

---

Signif. codes: 0 ‘***’ 0.001 ‘**’ 0.01 ‘*’ 0.05 ‘.’ 0.1 ‘ ’ 1

**Juveniles with osculum**

Anova for Shannon

Analysis of Variance Table

| Response: | invSimp |  |  |  |  |
| --- | --- | --- | --- | --- | --- |
|  | Df | Sum Sq | Mean Sq | F value | Pr(>F) |
| Date | 1 | 0.53 | 0.53 | 1.72 | 0.236 |
| Residuals | 6 | 1.85 | 0.3 |  |  |

---

Signif. codes: 0 ‘***’ 0.001 ‘**’ 0.01 ‘*’ 0.05 ‘.’ 0.1 ‘ ’ 1

Anova for InvSimp

Analysis of Variance Table

| Response: | invSimp |  |  |  |  |
| --- | --- | --- | --- | --- | --- |
|  | Df | Sum Sq | Mean Sq | F value | Pr(>F) |
| Date | 1 | 133.72 | 133.7 | 3.03 | 0.13 |
| Residuals | 6 | 264.4 | 44.08 |  |  |

---

Signif. codes: 0 ‘***’ 0.001 ‘**’ 0.01 ‘*’ 0.05 ‘.’ 0.1 ‘ ’ 1

**B. Beta diversity**

**Adults subset**

We test the effect of Individuals and replication within individuals (3 individual adults and 3 tissue replicates for each individual).

Permutest

Permutation test for homogeneity of multivariate dispersions

Permutation: free

Number of permutations: 999

| Response: | Distances | |  |  |  |  |
| --- | --- | --- | --- | --- | --- | --- |
|  | Df | Sum Sq | Mean Sq | F | N.Perm | Pr(>F) |
| Groups | 2 | 0.0053 | 0.0026 | 4.915 | 999 | **0.091** |
| Residuals | 6 | 0.0032 | 0.0005 |  |  |  |

PERMANOVA

Permutation test for adonis under reduced model

Terms added sequentially (first to last)

Permutation: free

Number of permutations: 999

adonis2(formula = counts_bc ~ Individual + Replicate, data = Infotable_AD)

|  | Df | SumOfSqs | R2 | F | Pr(>F) |
| --- | --- | --- | --- | --- | --- |
| Individual | 2 | 0.47 | 0.46 | 2.61 | **0.003**** |
| Replicate | 2 | 0.18 | 0.17 | 1 | 0.48 |
| Residual | 4 | 0.36 | 0.35 |  |  |
| Total | 8 | 1.02 | 1 |  |  |

---

Signif. codes: 0 ‘***’ 0.001 ‘**’ 0.01 ‘*’ 0.05 ‘.’ 0.1 ‘ ’ 1

**Free-living larvae subset**

We test the effect of Time for the free living larvae

Permutest

Permutation test for homogeneity of multivariate dispersions

Permutation: free

Number of permutations: 999

| Response: | Distances | |  |  |  |  |
| --- | --- | --- | --- | --- | --- | --- |
|  | Df | Sum Sq | Mean Sq | F | N.Perm | Pr(>F) |
| Groups | 1 | 5.4460e-08 | 5.4458e-08 | 0.78 | 999 | **0.425** |
| Residuals | 8 | 5.5614e-07 | 6.9517e-08 |  |  |  |

PERMANOVA

Permutation test for adonis under reduced model

Terms added sequentially (first to last)

Permutation: free

Number of permutations: 999

adonis2(formula = counts_bc ~ Date, data = Infotable_FL)

|  | Df | SumOfSqs | R2 | F | Pr(>F) |
| --- | --- | --- | --- | --- | --- |
| Date | 1 | 0.00003 | 0.75 | 25.3 | **0.01**** |
| Residual | 8 | 0.00001 | 0.24 |  |  |
| Total | 9 | 0.00005 | 1 |  |  |

---

Signif. codes: 0 ‘***’ 0.001 ‘**’ 0.01 ‘*’ 0.05 ‘.’ 0.1 ‘ ’ 1

**Juvenile with osculum subset**

We test the effect of Time for juveniles with osculum sampled at 2 time points

Permutest

Permutation test for homogeneity of multivariate dispersions

Permutation: free

Number of permutations: 999

| Response: | Distances | |  |  |  |  |
| --- | --- | --- | --- | --- | --- | --- |
|  | Df | Sum Sq | Mean Sq | F | N.Perm | Pr(>F) |
| Groups | 1 | 0.001 | 0.001 | 0.104 | 999 | **0.839** |
| Residuals | 6 | 0.058 | 0.09 |  |  |  |

PERMANOVA

Permutation test for adonis under reduced model

Terms added sequentially (first to last)

Permutation: free

Number of permutations: 999

adonis2(formula = counts_bc ~ Date, data = Infotable_LO)

|  | Df | SumOfSqs | R2 | F | Pr(>F) |
| --- | --- | --- | --- | --- | --- |
| Date | 1 | 0.60937 | 0.56788 | 7.885 | **0.021*** |
| Residual | 6 | 0.46369 | 0.43212 |  |  |
| Total | 7 | 1.07306 | 1 |  |  |

---

Signif. codes: 0 ‘***’ 0.001 ‘**’ 0.01 ‘*’ 0.05 ‘.’ 0.1 ‘ ’ 1

**All dataset**

We have considered 38 samples for this analysis. In the case of adult samples, first pseudoreplicates from the same individual were pooled together (mean abundance) and then analysis of variance and permanova were performed.

Permutest

Permutation test for homogeneity of multivariate dispersions

Permutation: free

Number of permutations: 999

| Response: | Distances | |  |  |  |  |
| --- | --- | --- | --- | --- | --- | --- |
|  | Df | Sum Sq | Mean Sq | F | N.Perm | Pr(>F) |
| Groups | 4 | 0.07 | 0.017 | 1.86 | 999.000 | **0.146** |
| Residuals | 33 | 0.31 | 0.009 |  |  |  |

PERMANOVA

Permutation test for adonis under reduced model

Terms added sequentially (first to last)

Permutation: free

Number of permutations: 1000

adonis2(formula = t(counts_RA_38) ~ Stage, data = Infotable_38, permutations = 1000, method = "bray")

|  | Df | SumOfSqs | R2 | F | Pr(>F) |
| --- | --- | --- | --- | --- | --- |
| Stage | 4 | 5.43 | 0.48 | 7.85 | **0.001***** |
| Residual | 33 | 5.7 | 0.51 |  |  |
| Total | 37 | 11.1 | 1 |  |  |

---

Signif. codes: 0 ‘***’ 0.001 ‘**’ 0.01 ‘*’ 0.05 ‘.’ 0.1 ‘ ’ 1

**C. Adults and Larvae subset**

**Bray-Curtis similarity**

Larvae offspring

Analysis of Variance Table

| Response: | value | |  |  |  |
| --- | --- | --- | --- | --- | --- |
|  | Df | Sum Sq | Mean Sq | F value | Pr(>F) |
| Stage | 1 | 0.020 | 0.019 | 6.49 | **0.012*** |
| Residuals | 154 | 0.369 | 0.003 |  |  |

---

Signif. codes: 0 ‘***’ 0.001 ‘**’ 0.01 ‘*’ 0.05 ‘.’ 0.1 ‘ ’ 1

Parents – offspring

Analysis of Variance Table

| Response: | value | |  |  |  |
| --- | --- | --- | --- | --- | --- |
|  | Df | Sum Sq | Mean Sq | F value | Pr(>F) |
| Stage | 1 | 0.105 | 0.104 | 27.6 | **3.3e-07 ***** |
| Residuals | 232 | 0.878 | 0.003 |  |  |

---

Signif. codes: 0 ‘***’ 0.001 ‘**’ 0.01 ‘*’ 0.05 ‘.’ 0.1 ‘ ’ 1

**Jaccard similarity**

Larvae offspring

Analysis of Variance Table

| Response: | value | |  |  |  |
| --- | --- | --- | --- | --- | --- |
|  | Df | Sum Sq | Mean Sq | F value | Pr(>F) |
| Stage | 1 | 0.010 | 0.011 | 6.89 | **0.0095 ***** |
| Residuals | 154 | 0.243 | 0.001 |  |  |

---

Signif. codes: 0 ‘***’ 0.001 ‘**’ 0.01 ‘*’ 0.05 ‘.’ 0.1 ‘ ’ 1

Parents – offspring

Analysis of Variance Table

| Response: | value | |  |  |  |
| --- | --- | --- | --- | --- | --- |
|  | Df | Sum Sq | Mean Sq | F value | Pr(>F) |
| Stage | 1 | 0.048 | 0.048 | 29.1 | **1.7e-07 ***** |
| Residuals | 232 | 0.384 | 0.001 |  |  |

---

Signif. codes: 0 ‘***’ 0.001 ‘**’ 0.01 ‘*’ 0.05 ‘.’ 0.1 ‘ ’ 1

**D. ASV1**

GLMM model for ASV1 abundances across stages with Individual nested within stage.

**Results of Anova:**

Analysis of Deviance Table (Type II Wald chisquare tests)

Response: ASV1

Chisq Df Pr(>Chisq)

Stage 206.89 4 < 2.2e-16 ***

---

Signif. codes: 0 ‘***’ 0.001 ‘**’ 0.01 ‘*’ 0.05 ‘.’ 0.1 ‘ ’ 1

**Pairwise comparisons:**

Degrees-of-freedom method: kenward-roger

Confidence level used: 0.95

| contrast | estimate | SE | df | t.ratio | p.value |
| --- | --- | --- | --- | --- | --- |
| AD-BL | -45.644 | 5.63 | 37.79 | -8.101 | <.0001 |
| AD-FL | -38.369 | 5.91 | 2.65 | -6.496 | 0.0386 |
| AD-JO | 26.388 | 6.25 | 3.3 | 4.224 | 0.0788 |
| AD-JNO | -39.321 | 7.72 | 7.38 | -5.09 | 0.0075 |
| BL-FL | 7.275 | 5.47 | 1.82 | 1.33 | NaN |
| BL-JO | 72.032 | 5.83 | 2.37 | 12.346 | 0.0113 |
| BL-JNO | 6.323 | 7.4 | 6.02 | 0.855 | 0.9035 |
| FL-JO | 64.757 | 6.1 | 37.02 | 10.62 | <.0001 |
| FL-JNO | -0.953 | 7.61 | 37.02 | -0.125 | 0.9999 |
| JO-JNO | -65.709 | 7.87 | 37.02 | -8.347 | <.0001 |

P value adjustment: tukey method for comparing a family of 5 estimates
