# Supplementary material for: Microbiome changes through the ontogeny of the marine sponge Crambe crambe
Source: Environ Microbiome. 2024 Mar 11;19:15. doi: 10.1186/s40793-024-00556-7 (PMC10929144; doi:10.1186/s40793-024-00556-7)
Supplement: Supplementary file 12 — Additional file 12: Table S1. Taxonomic composition at Phylum level for each ontogenetic stage of C. Crambe ordered in descending total abundance (last column). Numbers represent percentages (%) of average Relative abundances. Taxa with relative abundances < 0.01% are grouped as “Others”. AD: Adult, BL: Brooding Larvae, FL: Free Living larvae, JNO: Juvenile No Osculum, JO: Juvenile with Osculum, Total: mean relative abundance across all samples. [file 40793_2024_556_MOESM12_ESM.pdf]

Supplementary Table S1. Taxonomic composition at Phylum level for each ontogenetic stage ordered in descending total abundance (last column). Taxa with relative abundances < 0.01 are grouped as "Others". AD: Adult, BL: Brooding Larvae, FL: Free Living larvae, JNO: Juvenile No Osculum, JO: Juvenile with Osculum.

**Taxonomy**	**AD**	**BL**	**FL**	**JNO**	**JO**	**Total**
Bacteria;Proteobacteria	74.89	90.40	95.63	97.42	81.93	88.05
Bacteria;Bacteroidota	1.16	0.64	0.41	1.61	8.53	2.47
Bacteria;Cyanobacteria	9.51	0.84	0.12	0.02	0.03	2.10
Bacteria;Planctomycetota	3.47	1.09	0.23	0.00	1.03	1.17
Bacteria;Bacteria_unclassified	2.92	0.66	0.29	0.06	1.14	1.01
Archaea;Crenarchaeota	2.78	0.96	0.17	0.01	1.13	1.01
Bacteria;Firmicutes	0.15	1.92	1.04	0.01	0.72	0.77
Bacteria;Verrucomicrobiota	0.60	0.24	0.13	0.23	2.29	0.70
Bacteria;Actinobacteriota	0.90	0.80	0.44	0.03	0.15	0.46
Bacteria;Nitrospirota	1.15	0.41	0.07	0.00	0.08	0.34
Bacteria;Chloroflexi	0.37	0.36	0.24	0.01	0.53	0.30
Bacteria;Dependentiae	0.17	0.06	0.00	0.00	1.07	0.26
Bacteria;Acidobacteriota	0.25	0.35	0.13	0.00	0.44	0.24
Bacteria;Bdellovibrionota	0.02	0.03	0.72	0.27	0.05	0.22
Bacteria;Entotheonellaeota	0.63	0.24	0.04	0.00	0.05	0.19
Bacteria;Myxococcota	0.19	0.13	0.04	0.01	0.15	0.11
Bacteria;Dadabacteria	0.21	0.09	0.03	0.00	0.17	0.10
Bacteria;Deinococcota	0.05	0.43	0.00	0.00	0.00	0.09
Bacteria;Others	0.08	0.13	0.03	0.01	0.14	0.08
Bacteria;NB1-j	0.23	0.10	0.00	0.00	0.02	0.07
Bacteria;Desulfobacterota	0.24	0.03	0.02	0.04	0.02	0.07
Bacteria;Campylobacterota	0.00	0.01	0.04	0.26	0.01	0.06
Bacteria;PAUC34f	0.00	0.03	0.13	0.00	0.12	0.06
Bacteria;Gemmatimonadota	0.01	0.03	0.05	0.00	0.13	0.04
Bacteria;Patescibacteria	0.01	0.04	0.00	0.01	0.06	0.02
