# Supplementary material for: Microbiome changes through the ontogeny of the marine sponge Crambe crambe
Source: Environ Microbiome. 2024 Mar 11;19:15. doi: 10.1186/s40793-024-00556-7 (PMC10929144; doi:10.1186/s40793-024-00556-7)
Supplement: Supplementary file 13 — Additional file 13: Table S2. Taxonomic composition at Class level for each ontogenetic stage of C. crambe ordered in descending total abundance (last column). Numbers represent percentages (%) of average Relative abundances. Taxa with relative abundances < 0.01% are grouped as “Others”. AD: Adult, BL: Brooding Larvae, FL: Free Living larvae, JNO: Juvenile No Osculum, JO: Juvenile with Osculum, Total: mean relative abundance across all samples. In Taxonomy column, A: Archaea, B: Bacteria. [file 40793_2024_556_MOESM13_ESM.pdf]

Supplementary Table S2. Taxonomic composition at Class level for each ontogenetic stage ordered in descending total abundance (last column). Taxa with relative abundances < 0.01 are grouped as "Others". AD: Adult, BL: Brooding Larvae, FL: Free Living larvae, JNO: Juvenile No Osculum, JO: Juvenile with Osculum. In Taxonomy column, A: Archaea, B: Bacteria.

Taxonomy	AD	BL	FL	JNO	JO	Total
B;Proteobacteria;Gammaproteobacteria	61.79	87.48	94.44	93.05	68.34	81.02
B;Proteobacteria;Alphaproteobacteria	8.90	2.72	1.08	4.16	13.28	6.03
B;Bacteroidota;Bacteroidia	1.15	0.63	0.41	1.60	8.51	2.46
B;Cyanobacteria;Cyanobacteriia	9.48	0.80	0.11	0.02	0.01	2.09
B;Planctomycetota;Planctomycetes	3.18	0.91	0.23	0.00	0.75	1.01
B;Bacteria_unclassified;Bacteria_unclassified	2.92	0.66	0.29	0.06	1.14	1.01
A;Crenarchaeota;Nitrososphaeria	2.78	0.96	0.17	0.01	1.13	1.01
B;Proteobacteria;Proteobacteria_unclassified	4.20	0.20	0.11	0.21	0.31	1.01
B;Firmicutes;Bacilli	0.10	1.63	1.03	0.01	0.72	0.70
B;Verrucomicrobiota;Chlamydiae	0.20	0.04	0.00	0.00	1.91	0.43
B;Nitrospirota;Nitrospiria	1.15	0.41	0.07	0.00	0.08	0.34
B;Others	0.35	0.49	0.12	0.07	0.38	0.28
B;Dependentiae;Babeliae	0.17	0.06	0.00	0.00	1.07	0.26
B;Actinobacteriota;Actinobacteria	0.12	0.50	0.39	0.03	0.06	0.22
B;Bdellovibrionota;Bdellovibrionia	0.02	0.01	0.69	0.27	0.05	0.21
B;Entotheonellaeota;Entotheonellia	0.63	0.24	0.04	0.00	0.05	0.19
B;Actinobacteriota;Acidimicrobiia	0.65	0.16	0.05	0.00	0.08	0.19
B;Verrucomicrobiota;Verrucomicrobiae	0.34	0.13	0.12	0.01	0.30	0.18
B;Chloroflexi;Dehalococcoidia	0.01	0.11	0.18	0.00	0.38	0.14
B;Dadabacteria;Dadabacteriia	0.21	0.09	0.03	0.00	0.17	0.10
B;Acidobacteriota;Thermoanaerobaculia	0.18	0.19	0.02	0.00	0.08	0.09
B;Deinococcota;Deinococci	0.05	0.43	0.00	0.00	0.00	0.09
B;Planctomycetota;Phycisphaerae	0.19	0.09	0.00	0.00	0.16	0.09
B;Myxococcota;Polyangia	0.17	0.08	0.03	0.01	0.12	0.08
B;Verrucomicrobiota;Lentisphaeria	0.06	0.06	0.00	0.21	0.08	0.08
B;Chloroflexi;Anaerolineae	0.08	0.13	0.05	0.00	0.12	0.08
B;Acidobacteriota;Vicinamibacteria	0.03	0.09	0.06	0.00	0.18	0.07
B;NB1-j;NB1-j_cl	0.23	0.10	0.00	0.00	0.02	0.07
B;Campylobacterota;Campylobacteria	0.00	0.01	0.04	0.26	0.01	0.06
B;Chloroflexi;Chloroflexia	0.27	0.04	0.00	0.00	0.00	0.06
B;PAUC34f;PAUC34f_cl	0.00	0.03	0.13	0.00	0.12	0.06
B;Planctomycetota;OM190	0.07	0.06	0.00	0.00	0.11	0.05
B;Gemmatimonadota;BD2-11_terrestrial_group	0.01	0.02	0.05	0.00	0.13	0.04
B;Firmicutes;Thermoanaerobacteria	0.01	0.19	0.00	0.00	0.00	0.04
B;Desulfobacterota;Desulfovibrionia	0.16	0.01	0.00	0.00	0.00	0.03

B;Acidobacteriota;Subgroup_21	0.00	0.02	0.04	0.00	0.09	0.03
B;Firmicutes;Clostridia	0.04	0.09	0.01	0.00	0.00	0.03
B;Actinobacteriota;Thermoleophilia	0.07	0.03	0.00	0.00	0.01	0.02
B;Acidobacteriota;Acidobacteriae	0.01	0.03	0.00	0.00	0.07	0.02
B;Actinobacteriota;Rubrobacteria	0.01	0.09	0.00	0.00	0.00	0.02
