# Supplementary material for: Microbiome changes through the ontogeny of the marine sponge Crambe crambe
Source: Environ Microbiome. 2024 Mar 11;19:15. doi: 10.1186/s40793-024-00556-7 (PMC10929144; doi:10.1186/s40793-024-00556-7)
Supplement: Supplementary file 14 — Additional file 14: Table S3. Shannon diversity and InvSimpson index for each sample. Information on Individual, Replicate, Stage and Sampling date are also shown as separated columns. [file 40793_2024_556_MOESM14_ESM.pdf]

Supplementary Table S3. Shannon diversity and InvSimpson index for each sample. Information on Individual, Replicate, Stage and Sampling date are also shown as separated columns.

Original_label	Figures_label	Year	Individual	Replicate	Stage	Date	Shannon	InvSimp
Ccra_AD11_exp17	AD11	2017	ind1	rep1	Adult	August_09	3.51	6.17
Ccra_AD12_exp17	AD12	2017	ind1	rep2	Adult	August_09	3.69	6.83
Ccra_AD13_exp17	AD13	2017	ind1	rep3	Adult	August_09	3.76	6.45
Ccra_AD21_exp17	AD21	2017	ind2	rep1	Adult	August_09	3.30	5.27
Ccra_AD22_exp17	AD22	2017	ind2	rep2	Adult	August_09	3.33	4.37
Ccra_AD23_exp17	AD23	2017	ind2	rep3	Adult	August_09	3.74	5.53
Ccra_AD31_exp17	AD31	2017	ind3	rep1	Adult	August_09	4.13	7.46
Ccra_AD32_exp17	AD32	2017	ind3	rep2	Adult	August_09	3.18	4.86
Ccra_AD33_exp17	AD33	2017	ind3	rep3	Adult	August_09	3.82	6.26
Ccra_BL11_exp17	BL11	2017	ind1	rep1	BroodingLarvae	August_09	1.41	1.44
Ccra_BL12_exp17	BL12	2017	ind1	rep2	BroodingLarvae	August_09	1.28	1.43
Ccra_BL13_exp17	BL13	2017	ind1	rep3	BroodingLarvae	August_09	0.85	1.23
Ccra_BL21_exp17	BL21	2017	ind2	rep1	BroodingLarvae	August_09	1.33	1.45
Ccra_BL22_exp17	BL22	2017	ind2	rep2	BroodingLarvae	August_09	1.40	1.45
Ccra_BL23_exp17	BL23	2017	ind2	rep3	BroodingLarvae	August_09	0.78	1.21
Ccra_BL24_exp17	BL24	2017	ind2	rep4	BroodingLarvae	August_09	0.73	1.18
Ccra_BL25_exp17	BL25	2017	ind2	rep5	BroodingLarvae	August_09	1.14	1.32
Ccra_BL31_exp17	BL31	2017	ind3	rep1	BroodingLarvae	August_09	1.23	1.38
Ccra_BL32_exp17	BL32	2017	ind3	rep2	BroodingLarvae	August_09	2.14	1.87
Ccra_BL33_exp17	BL33	2017	ind3	rep3	BroodingLarvae	August_09	1.12	1.34

Ccra_BL34_exp17	BL34	2017	ind3	rep4	BroodingLarvae	August_09	2.57	2.61
Ccra_BL35_exp17	BL35	2017	ind3	rep5	BroodingLarvae	August_09	0.55	1.14
Ccra_FLL8_exp17	FLL8	2017	unk	rep8	LarvaeFreeLiving	August_09	0.39	1.11
Ccra_FLL9_exp17	FLL9	2017	unk	rep9	LarvaeFreeLiving	August_09	0.60	1.21
Ccra_FLL10_exp17	FLL10	2017	unk	rep10	LarvaeFreeLiving	August_09	0.41	1.11
Ccra_FLL11_exp17	FLL11	2017	unk	rep11	LarvaeFreeLiving	August_09	0.72	1.23
Ccra_FLL12_exp17	FLL12	2017	unk	rep12	LarvaeFreeLiving	August_09	0.43	1.16
Ccra_FLL1_exp17	FLL1	2017	unk	rep1	LarvaeFreeLiving	August_10	1.93	3.11
Ccra_FLL2_exp17	FLL2	2017	unk	rep2	LarvaeFreeLiving	August_10	1.69	3.04
Ccra_FLL3_exp17	FLL3	2017	unk	rep3	LarvaeFreeLiving	August_10	1.94	2.95
Ccra_FLL6_exp17	FLL6	2017	unk	rep6	LarvaeFreeLiving	August_10	1.14	1.77
Ccra_FLL7_exp17	FLL7	2017	unk	rep7	LarvaeFreeLiving	August_10	1.31	1.92
Ccra_SO1a_exp17	JNO1a	2017	unk	rep1	JuvenileWithOutOsculum	August_17	0.12	1.04
Ccra_SO1b_exp17	JNO1b	2017	unk	rep2	JuvenileWithOutOsculum	August_17	1.32	1.58
Ccra_SO1c_exp17	JNO1c	2017	unk	rep3	JuvenileWithOutOsculum	August_17	1.19	1.61
Ccra_SO1d_exp17	JNO1d	2017	unk	rep4	JuvenileWithOutOsculum	August_17	2.56	3.14
Ccra_CO2e_exp17	JO2e	2017	unk	rep5	JuvenileWithOsculum	August_19	4.00	9.38
Ccra_CO2f_exp17	JO2f	2017	unk	rep6	JuvenileWithOsculum	August_19	3.63	8.90
Ccra_CO2g_exp17	JO2g	2017	unk	rep7	JuvenileWithOsculum	August_19	3.02	6.10
Ccra_CO2h_exp17	JO2h	2017	unk	rep8	JuvenileWithOsculum	August_19	3.61	7.41
Ccra_CO2a_exp17	JO2a	2017	unk	rep1	JuvenileWithOsculum	August_31	4.31	24.07
Ccra_CO2b_exp17	JO2b	2017	unk	rep2	JuvenileWithOsculum	August_31	4.01	11.47
Ccra_CO2c_exp17	JO2c	2017	unk	rep3	JuvenileWithOsculum	August_31	4.75	23.91
Ccra_CO2d_exp17	JO2d	2017	unk	rep4	JuvenileWithOsculum	August_31	3.17	5.16
