# Supplementary material for: Microbiome changes through the ontogeny of the marine sponge Crambe crambe
Source: Environ Microbiome. 2024 Mar 11;19:15. doi: 10.1186/s40793-024-00556-7 (PMC10929144; doi:10.1186/s40793-024-00556-7)
Supplement: Supplementary file 15 — Additional file 15:Table S4. Microbial core communities of each developmental stage of C. crambe. Core communities are calculated based on the number of ASVs found in 100% and 70% of the total number of replicates in each stage (columns 4 and 5, respectively). Percentages indicate the average relative abundance of the core ASVs in each stage. In grey, the values used for comparisons in the Upset plot. [file 40793_2024_556_MOESM15_ESM.pdf]

Table S4. Microbial core communities of each developmental stage of *C. crambe.* Core communities are calculated based on the number of ASVs found in 100% and 70% of the total number of replicates in each stage (columns 4 and 5, respectively). Percentages indicate the average relative abundance of the core ASVs in each stage. In grey, the values used for comparisons in the Upset plot.

Stage	Num. replicates	Total ASVs	CORE 100%/ (%relab)	CORE 70% / (%relab)
**Adult (AD)**	9	10923	104/ 78.8%	210/ 82.4%
**Brooding Larvae (BL)**	13	5369	13 / 86.2%	40 / 88.4%
**Free Living (FL)**	10	1164	2 / 77.1%	4 / 77.4%
FL1 (09 Aug)	5	374	2 / 93.3%	3 / 93.3%
FL2 (10 Aug)	5	845	55 / 96.3%	83 / 96.7%
**Juvenile without osculum (JNO)**	4	1067	13 / 85.3%	96 / 93.3%
**Juvenile with osculum (JO)**	8	5681	77 / 50.6%	221 / 61.87%
JO1 (19 Aug)	4	2991	228/ 87.4 %	445 / 91.5%
JO2 (31 Aug)	4	3579	190/ 82.1 %	432 / 89.6 %
