# Supplementary material for: Microbiome changes through the ontogeny of the marine sponge Crambe crambe
Source: Environ Microbiome. 2024 Mar 11;19:15. doi: 10.1186/s40793-024-00556-7 (PMC10929144; doi:10.1186/s40793-024-00556-7)
Supplement: Supplementary file 17 — Additional file 17: Table S6. Differential Abundance (DA) analysis between the different ontogenetic stages for C. crambe. In grey we show the comparisons that appear in the Fig. 6. In the case of Free-Living larvae (FL) and Juveniles with Osculum (JO), when the different times are considered they are shown as FL1 (09 Aug), FL2 (10 Aug) and JO1 (19 Aug) and JO2 (31 Aug). [file 40793_2024_556_MOESM17_ESM.pdf]

Table S6. Differential Abundance (DA) analysis between the different ontogenetic stages for *C. crambe.* In grey we show the comparisons that appear in the Figure 6. In the case of Free-Living larvae (FL) and Juveniles with Osculum (JO), when the different times are considered they are shown as FL1 (09 Aug), FL2 (10 Aug) and JO1 (19 Aug) and JO2 (31 Aug).

Comparison	n. replicates	N. ASV used	Stage	DA	DA total
ADULT vs BroodingLarvae	9 AD vs 13 BL	710	AD	96	116
			BL	20	
BroodingLarvae vs FreeLiving	13 BL vs 10 FL	701	BL	77	101
			FL	24	
BroodingLarvae vs FreeLiving1	13 BL vs 5 FL1	641	BL	16	18
			FL	2	
BroodingLarvae vs FreeLiving2	13 BL vs 5 FL	547	BL	28	71
			FL	43	
FreeLiving1 vs FreeLiving2	5 FL vs 5 FL	609	FL1	0	42
			FL2	42	
FreeLiving vs Juvenile No osc	10 FL vs 4 JNO	349	FL	0	88
			JNO	88	
FreeLiving 1 vs Juvenile No osc	5 FL1 vs 4 JNO	288	FL1	0	29
			JNO	29	
FreeLiving 2 vs Juvenile No osc	5 FL2 vs 4 JNO	180	FL2	38	91
			JNO	53	
Juvenile No osc vs Juvenile Osc	4 JNO vs 8 JO	408	JNO	60	180
			JO	120	
Juvenile No osc vs Juvenile Osc1	4 JNO vs 4 JO1	302	JNO	38	187
			JO1	149	
Juvenile No osc vs Juvenile Osc2	4 JNO vs 4 JO2	312	JNO	29	133
			JO2	104	
Juvenile Osc1 vs Juvenile Osc2	4 JO1 vs 4 JO2	332	JO1	53	91
			JO2	38	
Juvenile Osc vs Adult	9 AD vs 8 JO	526	JO	123	300
			AD	177	
Juvenile Osc1 vs Adult	9 AD vs 4 JO1	401	JO1	160	250
			AD	90	

Comparison	n. replicates	N. ASV used	Stage	DA	DA total
Juvenile Osc2<br>vs<br>Adult	9 AD vs 4 JO2	431	JO2	145	261
			AD	116	
